# Immunophenotype and Transcriptome Profile of Patients With Multiple Sclerosis Treated With Fingolimod: Setting Up a Model for Prediction of Response in a 2-Year Translational Study

**DOI:** 10.3389/fimmu.2018.01693

**Published:** 2018-07-25

**Authors:** Irene Moreno-Torres, Coral González-García, Marco Marconi, Aranzazu García-Grande, Luis Rodríguez-Esparragoza, Víctor Elvira, Elvira Ramil, Lucía Campos-Ruíz, Ruth García-Hernández, Fátima Al-Shahrour, Coral Fustero-Torre, Alicia Sánchez-Sanz, Antonio García-Merino, Antonio José Sánchez López

**Affiliations:** ^1^Neuroimmunology Unit, Puerta de Hierro-Segovia de Arana Health Research Institute, Madrid, Spain; ^2^Autonomous University of Madrid, Madrid, Spain; ^3^Centre for Plant Biotechnology and Genomics, Madrid, Spain; ^4^Flow Cytometry Core Facility, Puerta de Hierro-Segovia de Arana Health Research Institute, Madrid, Spain; ^5^Spanish National Cardiovascular Research Center (CNIC), Madrid, Spain; ^6^IMT Lille Douai & CRIStAL, Univ. de Lille, Douai, France; ^7^Sequencing Core Facility, Puerta de Hierro-Segovia de Arana Health Research Institute, Madrid, Spain; ^8^Bioinformatics Unit of Spanish National Cancer Research Center (CNIO), Madrid, Spain; ^9^Neurology Department, Puerta de Hierro University Hospital, Madrid, Spain; ^10^Red Española de Esclerosis Múltiple (REEM), Barcelona, Spain; ^11^Biobank, Puerta de Hierro University Hospital-IDIPHISA, Madrid, Spain

**Keywords:** multiple sclerosis, biomarkers, fingolimod, lymphocyte subpopulations, RNA-seq, transcriptome

## Abstract

**Background:**

Fingolimod is a functional sphingosine-1-phosphate antagonist approved for the treatment of multiple sclerosis (MS). Fingolimod affects lymphocyte subpopulations and regulates gene expression in the lymphocyte transcriptome. Translational studies are necessary to identify cellular and molecular biomarkers that might be used to predict the clinical response to the drug. In MS patients, we aimed to clarify the differential effects of fingolimod on T, B, and natural killer (NK) cell subsets and to identify differentially expressed genes in responders and non-responders (NRs) to treatment.

**Materials and methods:**

Samples were obtained from relapsing–remitting multiple sclerosis patients before and 6 months after starting fingolimod. Forty-eight lymphocyte subpopulations were measured by flow cytometry based on surface and intracellular marker analysis. Transcriptome sequencing by next-generation technologies was used to define the gene expression profiling in lymphocytes at the same time points. NEDA-3 (no evidence of disease activity) and NEDA-4 scores were measured for all patients at 1 and 2 years after beginning fingolimod treatment to investigate an association with cellular and molecular characteristics.

**Results:**

Fingolimod affects practically all lymphocyte subpopulations and exerts a strong effect on genetic transcription switching toward an anti-inflammatory and antioxidant response. Fingolimod induces a differential effect in lymphocyte subpopulations after 6 months of treatment in responder and NR patients. Patients who achieved a good response to the drug compared to NR patients exhibited higher percentages of NK bright cells and plasmablasts, higher levels of FOXP3, glucose phosphate isomerase, lower levels of FCRL1, and lower Expanded Disability Status Scale at baseline. The combination of these possible markers enabled us to build a probabilistic linear model to predict the clinical response to fingolimod.

**Conclusion:**

MS patients responsive to fingolimod exhibit a recognizable distribution of lymphocyte subpopulations and a different pretreatment gene expression signature that might be useful as a biomarker.

## Introduction

Multiple sclerosis (MS) is a chronic inflammatory demyelinating disease of the central nervous system (CNS). MS is characterized by an autoimmune response against CNS myelin, infiltration of the brain and spinal cord by inflammatory cells and axonal damage. In recent years, a better understanding of the immunologic mechanisms implicated in the destruction of CNS components in MS has led to the effective design of new therapies. It has been shown that activation of encephalitogenic T and B cells, which are implicated in neuronal damage, occurs primarily outside the CNS, and the migration of these activated cells from lymph nodes to peripheral blood plays a central role in the pathogenesis of MS (1). In addition, it is known that MS patients have a higher number of autoreactive T and B cells as well as fewer regulatory T cells (Tregs) (2) and B1 cells (3) than healthy controls (HC) in peripheral blood. Such therapies as fingolimod (Gilenya^®^), a functional sphingosine-1-phosphate (S1P) antagonist, have been developed to retain the autoreactive lymphocytes within the lymph nodes (4, 5). The phosphorylated form of fingolimod binds to four of five S1P-receptors (S1P_1_ and S1P_3–5_), resulting in aberrant internalization and degradation of the receptor on the cell surface and blockade of the egress and recirculation of activated CCR7+ and CD62+ lymphocytes from lymph nodes, such as central memory (TCM) and naïve T (TN) cells. In addition, S1P receptors are differentially expressed in several tissues and have significant roles in a variety of cellular responses, including survival, inhibition of apoptosis, cardioprotection, activation of innate and adaptive immune systems, Treg differentiation, and promoting Th1 and Th17 differentiation *in vitro* and *in vivo* (6). These phenomena could explain the relative increase in Tregs and the reduction of Th1 and Th17 cells in response to fingolimod (7). In addition, an increase in the percentage of B cells with regulatory functions has also been observed in treated patients (8).

The assessment of the effectiveness of treatment for MS is complex and requires the use of clinical and imaging criteria such as the annualized relapse rate (ARR), the cumulative progression of disability, and the number and volume of new T2 or gadolinium-enhanced lesions. NEDA-3 (no evidence of disease activity) is a composite measure of disease activity in MS [including relapses, disability progression, and magnetic resonance imaging (MRI) activity] that is used as a secondary outcome measure in clinical trials for new disease-modifying therapies (DMTs) (9). These measures are focused on the inflammatory process and do not take into account other important aspects of MS pathophysiology such as neurodegeneration. Recently, a fourth measure, brain volume loss, has been suggested to be added to NEDA because of its relation to cognitive decline in MS patients. This new composite measure is called NEDA-4 and has advantages over the conventional NEDA-3 for predicting subsequent disability and structural damage (10). Despite efforts, the use of response biomarkers at the clinical level is limited, and studies designed to identify patients who require an exhaustive follow-up are necessary.

To search for biomarkers of response and to improve our understanding of mechanisms of action of fingolimod, we carried out a translational study of the cellular and transcriptional characteristics of MS patients treated with fingolimod. Patients were classified according to the clinical response which was measured by the NEDA-3 and NEDA-4 status at 1 and 2 years, and samples were analyzed by flow cytometry immunophenotyping and RNA next-generation sequencing before and after 6 months of treatment. The possible markers obtained by a first statistical approach were used to build a model of prediction that might be useful to anticipate the clinical response to fingolimod.

## Materials and Methods

### Patients, Control Subjects, and Samples

Enrollment was limited to patients 18–55 years of age with a diagnosis of relapsing–remitting multiple sclerosis (RRMS) according to McDonald 2010 criteria (11) with an indication for treatment with fingolimod and an entry score of 0–5.0 in the Expanded Disability Status Scale (EDSS). MS patients categorized as progressive or previously treated with an S1P receptor agonist were excluded. A total of 40 patients were recruited, and all of them signed an informed consent that was previously approved by the Ethics Committee of Puerta de Hierro University Hospital. The patients were treated with fingolimod 0.5 mg once daily, according to the current recommendations of use for fingolimod. The treated patients were subdivided into two subgroups according to the previous use of natalizumab (NTZ): NTZ group (*n* = 18) and no-NTZ group (*n* = 22) because switching from NTZ has been associated with a risk of MS reactivation (12). The patients of NTZ-group started fingolimod treatment after a washout period of 1–2 months and in patients of No-NTZ group (4 patients from glatiramer acetate and 18 patients from IFN-β), no washout period was needed according to the current guidelines. Venous blood samples were obtained from patients immediately before starting treatment with fingolimod and 6 months after starting therapy.

Samples were also obtained from 10 HC and 10 naïve MS patients (who had never received any disease-modifying treatment) matched for age and sex with treated individuals. The demographic characteristics of the patients and controls and baseline differences between subgroups are shown in Table 1.

**Table 1:** Demographic characteristics of patients and control subjects.

Cytometry experiments							
	Groups			Subgroups			
	Patients (*n* = 40)	Naive MS (*n* = 10)	HC (*n* = 10)	NTZ Group (*n* = 18) 45%	No-NTZ Group (*n* = 22) 55%		
Age (years)*	40.63 ± 9.67	36.1 ± 8.33	37 ± 9.27	43.06 ± 9.43	39 ± 8.42		
Sex (% of female)^~^	25 (62.5%)	6 (60%)	6 (60%)	10 (55.5%)	15 (68.18%)		
Disease duration (years)*^,^ ^†^	12.2 ± 6.39^ 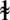 ^	5.6 ± 2.50^ 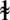 ^	–	16.33 ± 6.03^ 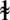 ^	8.82 ± 4.45^ 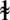 ^		
Time since DMT onset (years)*	9.58 ± 5.58	–	–	12.89 ± 4.95^ 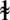 ^	6.86 ± 4.58^ 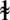 ^		
Number of previous DMTs (years)*	1.95 ± 1.06	–	–	2.78 ± 0.73^ 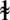 ^	1.27 ± 0.77^ 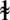 ^		
Basal ARR*^,^ ^‡^	0.53 ± 0.72	0.94 ± 0.56	–	0.06 ± 0.24^ 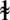 ^	0.91 ± 0.75^ 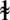 ^		
Basal EDSS*	2.63 ± 1.59^ 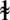 ^	0.60 ± 0.73^ 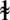 ^	–	3.17 ± 1.68	2.18 ± 1.4		
Number of GdE lesions*	0.38 ± 1.03^ 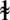 ^	0.80 ± 1.01^ 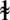 ^	–	0 ± 0^ 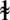 ^	0.68 ± 1.32^ 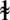 ^		
GdE lesion volume (cm^3^)*	0.27 ± 0.89	0.42 ± 0.74	–	0 ± 0^ 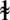 ^	0.49 ± 1.16^ 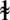 ^		
Number of new T2-Weighted lesions*^,^ ^¶^	0.45 ± 0.99	–	–	0.22 ± 0.73	0.64 ± 1.14		
T2-weighted lesion volume (cm^3^)*	9.52 ± 7.17	5.15 ± 4.10	–	9.46 ± 7.97	9.56 ± 6.64		
T1-weighted lesion volume (cm^3^)*	1.06 ± 1.36^ 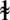 ^	0.24 ± 0.41^ 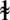 ^	–	1.44 ± 1.78	0.75 ± 0.81		

**RNA-seq experiments**							

	**Groups**			**Subgroups**			
	**Patients (*n* = 10)**	**Naive MS (*n* = 5)**	**HC (*n* = 5)**	**Responder 1 year (*n* = 5)**	**Non-responder (NR) 1 year (*n* = 5)**	**Responder 2 years (*n* = 4)**	**NR 2 years (*n* = 6)**

Age (years)*	40.3 ± 11.06	34.2 ± 10.75	32.8 ± 6.2	43.2 ± 10.6	37.4 ± 11.8	45 ± 11.4	37.2 ± 10.6
Sex (% of female)^~^	6 (60%)	3 (60%)	2 (50%)	2 (40%)	4 (80%)	2 (50%)	4 (67%)
Disease duration (years)*^,^ ^†^	11 ± 5.96^ 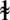 ^	4.6 ± 2.79^ 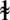 ^	–	13.8 ± 6.1	8.20 ± 4.7	15.7 ± 4.99^ 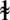 ^	7.83 ± 4.35^ 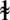 ^
Time since DMT onset (years)*	9.4 ± 6.04	–	–	12.6 ± 5.7	6.2 ± 4.9	14.25 ± 4.99^ 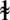 ^	6.17 ± 4.4^ 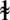 ^
Number of previous DMTs (years)*	2 ± 0.94	–	–	2.4 ± 0.5	1.6 ± 1.1	2.5 ± 0.6	1.7 ± 1.03
Basal ARR*^,^ ^‡^	0.70 ± 0.82	1 ± 0.70	–	0.2 ± 0.44^ 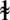 ^	1.2 ± 0.8^ 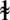 ^	0 ± 0^ 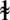 ^	1.16 ± 0.75^ 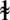 ^
Basal EDSS*	2.25 ± 1.7^ 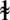 ^	0.7 ± 0.67^ 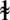 ^	–	2.1 ± 2.3	2.4 ± 0.89	2.62 ± 2.28	2 ± 1.26
Number of GdE lesions*	0.1 ± 0.31^ 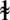 ^	0.8 ± 0.83^ 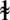 ^	–	0 ± 0	0.20 ± 0.44	0 ± 0	1.16 ± 0.40
GdE lesion volume (cm^3^)*	0.036 ± 0.11	0.21 ± 0.24	–	0 ± 0	0.07 ± 0.16	0 ± 0	0.06 ± 0.14
Number of new T2-Weighted lesions*^,^ ^¶^	0.1 ± 0.31	–	–	0 ± 0	0.20 ± 0.44	0 ± 0	0.16 ± 0.40
T2-weighted lesion volume (cm^3^)*	7.29 ± 6.86	7.21 ± 4.39	–	9.45 ± 8.94	5.12 ± 3.8	8.84 ± 10.21	6.25 ± 4.36
T1-weighted lesion volume (cm^3^)*	0.81 ± 1.04	0.50 ± 0.51	–	1 ± 1.35	0.62 ± 0.73	1.21 ± 1.5	0.55 ± 0.68

*Samples from all patients were used for cytometry experiments. 10 samples of patients, 5 samples of naïve MS, and 5 samples of HC were used for RNA-seq experiments*.

*The values are the mean ± SD of each group.

*^
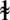
^Significant differences between groups or subgroups. A *p*-value <0.05 was considered statistically significant. The *p*-values were calculated using the Mann–Whitney test for differences between groups (Patients vs Naïve MS and Patients vs HC) and subgroups (NTZ vs No-NTZ or responder vs NR)*.

*^~^The value is the percentage of women in each group, and a Chi-square test was used to compare two proportions*.

*^†^Disease duration means the time since the first symptom of MS*.

*^‡^For basal ARR, only the last year was considered*.

*^¶^Data not available for MS controls, as most of them had a single magnetic resonance imaging (MRI). As expected, naïve MS patients showed a significantly shorter disease duration and lesser EDSS and MRI activity than study patients. Patients in the NTZ-group were significantly older, had a longer disease duration, greater number of previous treatments, and lower clinical and MRI activity than patients in the No-NTZ group because NTZ is a more efficacious drug than first-line medications. Responder patients had a lesser ARR and shorter treatment time with DMTs*.

*MS, multiple sclerosis; ARR, annualized relapse rate; DMTs, disease-modifying therapies; EDSS, Expanded Disability Status Scale; GdE, gadolinium-enhanced T1 lesions; HC, healthy controls*.

### MRI Measures and Clinical Response

Expanded Disability Status Scale, clinical relapses, and ARR were evaluated in all patients at baseline, 12 and 24 months after starting therapy. At the same times, a 1.5 T brain MRI was performed. The measured variables were as follows: number and volume of gadolinium-enhanced T1 lesions (GdE), number of new or enlarged T2-weighted lesions (T2w), total T2 and T1-weighted lesion volume, and annual brain volume loss (BVL), which was obtained using the SIENA method. The percentage of patients with MRI activity measured by the appearance of new GdE and/or T2w lesions was calculated. Confirmed disability progression (CDP) was defined by an increase of at least 1 EDSS point sustained during 1 year. NEDA-3 was calculated according to published parameters (no MRI activity, no relapses, and no CDP). NEDA-4 was calculated as NEDA-3 plus BVL <0.4%.

The patients who achieved NEDA-4 status were defined as responders at 1 or 2 years and the patient who did not achieve it were defined as non-responders (NRs) at 1 or 2 years.

### Antibodies and Reagents for Flow Cytometry

Peripheral blood mononuclear cells (PBMCs) were obtained from all blood samples by centrifugation on a Ficoll–Hypaque gradient and frozen in liquid nitrogen until use. For surface and intracellular staining, PBMCs were incubated using seven combinations of the fluorochrome-conjugated antibodies (FCAs) to define 48 lymphocyte subpopulations of B, T, and natural killer (NK) cells, as shown in Table S1 in Supplementary Material.

For intracellular staining, lymphocytes were stimulated for 4 h with phorbol 12-myristate 13-acetate (100 ng/ml) and ionomycin Io (1 µg/ml) in the presence of brefeldin A (10 µg/ml) from Sigma (St. Louis, MI, USA). The cells were stained with FCAs for surface markers before fixation and permeabilization with the specific INTRACELL-Kit (Immunostep). Fluorochrome-conjugated isotypes matched Abs were used as controls. All FCAs were obtained from Miltenyi Biotec (Auburn, CA, USA). The lymphocyte subpopulations and cytokine profiles were analyzed using a MACSQUANT flow cytometer (Miltenyi Biotec) and MACSQuantify 2.5 and FlowJo (tree Star) software. The gating strategy is shown in Figures S1.1–S1.7 in Supplementary Material.

### Total RNA Isolation and cDNA Library Preparation for Transcriptome Sequencing (RNA-seq)

Once the NEDA-4 status was determined in all patients, 10 of them were selected according to clinical response, five responders and five NRs at 1 year. In regard to response at 2 years, four of them were responders and six NRs. PBMCs obtained from these patients before and at 6 months of treatment were used to RNA sequencing as well as the samples from five naïve MS patients and five HC. The demographic characteristics of these patients and controls in this subset were similar to the whole sample and are shown in Table 1, as well as the baseline differences between responder and NR patients.

Total RNA was extracted using the Maxwell^®^ 16 LEV simply RNA Cells kit performed on Promega’s robotic platform according to the manufacturer’s instructions. The polyA+ fraction was purified and randomly fragmented, converted to double-stranded cDNA and processed through subsequent enzymatic treatments of end-repair, dA-tailing, and ligation to adapters as in Illumina’s “TruSeq Stranded mRNA Sample Preparation Part # 15031047 Rev. D” kit. The adapter-ligated library was completed by PCR with Illumina PE primers. The resulting purified cDNA library was applied to an Illumina flow cell for cluster generation and sequenced on an Illumina HiSeq2500 by following the manufacturer’s protocols. Image analysis, per-cycle base calling, and quality score assignment were performed with Illumina Real Time Analysis software. Conversion of Illumina BCL files to bam format was performed with the Illumina2bam tool (Wellcome Trust Sanger Institute—NPG). Unaligned single read sequences are provided in BAM files. Read qualities are provided in the standard Qscore Sanger encoding (Illumina 1.9, ASCII offset of 33).

### Statistic Analysis

A logistic regression model was fitted to the lymphocyte subpopulations data to determine the relative importance of each population as a predictor of response for the variables of interest (NEDA3, NEDA-4, MRI activity, clinical activity, and CDP). The model was fitted with the rstanarm package (13). The mean of each posterior distribution was used to create the heatmap with the pheatmap package (14). Four variables from the first approach were selected to first construct a two-dimensional data representation by nonparametric multidimensional scaling (Seuclidean method) and then to visualize differences between responder and NR subgroups. Spearman correlations (rho) for pairs of subpopulations were assessed at baseline and 6 months.

### Bioinformatics Analysis

To assess the effect of fingolimod on PBMCs from MS patients, the differentially expressed genes (DEGs) of samples before and after 6 months of treatment were analyzed. In samples collected at 6 months, DEGs between responder and NR patients were determined to analyze the differential effect of fingolimod according to the clinical response. To search for possible predictor genes of a clinical response, we analyzed DEGs between responder and NR patients using samples that were collected before treatment. The sequencing quality was analyzed with FastQC (15). Reads were aligned to the human genome (GRch38/*hg38*) (16) using STAR (17) and Samtools 0.1.19.0 (18), and transcript assembly, abundance estimation and differential expression were calculated with DESeq2 (19). The estimated *p*-value was corrected to account for multiple hypothesis testing using Benjamini and Hochberg false discovery rate (FDR) adjustment. Genes with an FDR less than or equal to 0.05 were considered differentially expressed.

In addition, we conducted a gene set enrichment analysis (GSEA), a publicly available computational method that employs pre-defined sets of genes to identify statistically significant differences between two biological conditions. This method can detect the upregulation or downregulation of recognized cellular pathways and biological processes under experimental conditions. GSEA was performed using GSEA software v2.2.1 obtained from the Broad Institute (20) and following the developer’s protocol (21). The previously obtained DEGs were ranked according to their *t*-statistics. This ranked file was used as input for the enrichment analysis. All basic and advanced fields were set to default and only those gene sets significantly enriched at an FDR *q*-values <0.25 were considered. To investigate how selected pathways were modulated by fingolimod therapy, we used the models from the wikipaths platform (22) and drew the differential regulation between responder and NR patients using the PathVisio program (23).

### Quantitative PCR (qPCR) Validation

Based on the RNA-seq analysis, we selected 10 significant DEGs for validation using qPCR analysis. Each RNA sample (500 ng) was reverse-transcribed using the NZY^®^ First-Strand cDNA Synthesis Kit. qPCR was performed in an LC480-Roche. The reaction conditions consisted of 6 µl cDNA and 1 µl of a 5 µM forward and reverse primer mix (Table S2 in Supplementary Material) and 5 µl of Light Cycler ^®^SYBR green I Master Mix.

Each sample was analyzed in duplicate for each individual reaction. The PCR program consisted of denaturation at 95°C for 5 min, 45 cycles of denaturing for 10 s at 95°C, annealing for 10 s at 60°C and extension for 10 s. A melting curve was generated by denaturation for 5 s at 95°C, annealing for 1 min at 65°C, and a ramp rate of 0.11°C/s up to 97°C. The gene expression of three endogenous genes (Table S2 in Supplementary Material) was used to normalize the target gene expression, and relative quantification was established using the 2^−ΔΔCt^ method (24).

### Design of Predictive Model

From the cytometry and RNA-seq experiments, variables that showed statistically significant differences between responder and NR were selected. To provide a tool for prediction of response to fingolimod, we fitted a probabilistic linear model to selected data and evaluated the resulting predictions defined as follows:

$$Y_{i}=x_{i}^{t}\;\text{β}+{\text{ε}}_{i}.$$

The model is based on the Bernoulli distribution or dichotomous distribution, in which the dependent variable can take the value *Y_i_* = 1 for the probability of success (responder to 2 years) or *Y_i_* = 0 for the probability of failure (NR to 2 years). The distribution of the sample is located in a cloud of points in such a way that their location divides the observations into two groups according to response. The calculated value of the regression line measures the response probability. Values close to 0 have a low probability of occurrence, while values close to 1 indicate that there is a high probability of a good response to treatment.

### Validation of Model

The expression levels by qPCR of genes used in the model building were measured on the pretreatment samples obtained from the remaining 30 patients who were not used for the analysis of RNA-seq. All of them have available data on the clinical evolution up to 2 years (NEDA-4) and results of cytometry experiments before treatment. To evaluate out-of-sample prediction, we computed the exact leave-one-out cross-validation (25).

## Results

### Clinical and MRI Response

**Figure 1:**
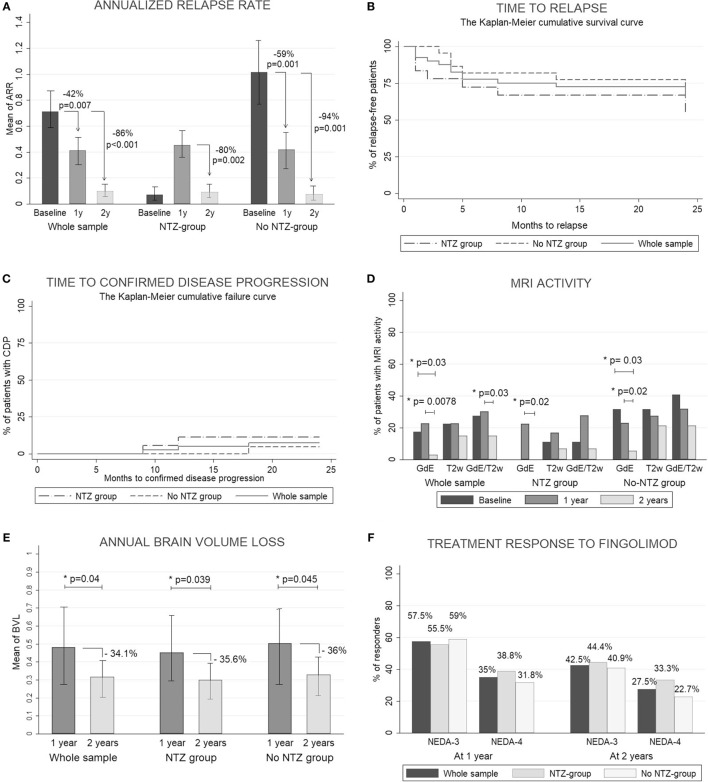
Clinical and radiological response. **(A)** Count data for annualized relapse rate (ARR) were fitted to a zero-inflated Poisson model. Estimated ARRs were calculated at different time periods (baseline, 1 year, and 2 years). To assess the statistical significance between groups, we obtained the *p*-values by calculating the probability of observing a group rate given the other group rate as the null hypothesis. **(B)** Kaplan–Meier cumulative survival curve for time to relapse. **(C)** Kaplan–Meier cumulative failure curve for time to confirmed disease progression. **(D)** Percentage of patients with magnetic resonance imaging (MRI) activity measured by the development of new gadolinium-enhanced T1 lesions (GdE), new T2-weighted lesions (T2w), or any MRI activity: development of new GdE lesions and/or T2w lesions (GdE/T2w). All measures were acquired at 1 and 2 years. The McNemar test was used to compare percentages between baseline and 1 and 2 years. **(E)** The Wilcoxon signed-rank test was used to compare the mean of the annual brain volume loss (BVL) between 1 and 2 years (measured by SIENA method). **(F)** Percentage of patients with a good response to fingolimod according NEDA-3 (no evidence of disease activity: no relapses, no any MRI activity and no clinical disease progression) and NEDA-4 (like NEDA but including BVL < 0.4). *p* < 0.05 was considered statistically significant.

In this study, fingolimod significantly reduced the mean ARR in 47.2 and 88.6% at 1 and 2 years, respectively, and the percentage of relapse-free patients was ~75% at the end of the study without differences between the subgroups. The percentage of patients with CDP was less than 10% at 1 and 2 years. Fingolimod reduced the percentage of patients with MRI activity at 2 years. A transitory increase in the ARR and the number of GdE lesions in the NTZ group was observed through the first year, but at 2 years these variables were decreased with no differences between the subgroups. There were no differences in the mean EDSS and number of new T2-w lesions at 1 and 2 years. At 2 years, the mean annual BVL was lower than at 1 year with no significant differences between the subgroups. At 1 year, the percentage of responder patients was 57.5% (*n* = 23) and 35% (*n* = 14) according to NEDA-3 and NEDA-4, respectively. At 2 years, it was 42.5% (*n* = 17) and 27.5% (*n* = 11) without differences between the subgroups. The results obtained at 1 and 2 years as well as all clinical and MRI measures are shown in Figures 1A–F and Table S3 in Supplementary Material. The treatment was discontinued in six patients, one because of adverse events and five due to a lack of efficacy. All of these patients were classified as NRs at 2 years in an intention-to-treat analysis.

### Characterization of Lymphocyte Subpopulations in MS Patients Before and After Fingolimod Treatment

In lymphocyte populations, no significant differences between HC and patients were observed before treatment, except for the percentage of LB1 cells composed mostly of CD11b+ cells, which was smaller in patients. At baseline, the only difference between subgroups was a higher percentage of transitional B cells in the NTZ-group compared with the no-NTZ-group (Tables S4 and S5 in Supplementary Material).

We could verify that fingolimod affected practically all lymphocyte populations and subpopulations of B, T, and NK cells. As expected, a reduction of the percentages of CD3+, CD4+, CD20+, CD19+, TCM and TN, memory B (switched and no-switched), regulatory B, NK bright, and cytokine-producing cells (IFN, IL-17, and IL-2) was observed after treatment (*p* < 0.001 for all), with a relative increase in effector memory T (TEM), terminally differentiated effector T (TEMRA), NK, NK dim, NKT, Tregs, naïve B, immature B, transitional B, CD5+ B, LB1, and plasmablast cells (*p* < 0.001 for all) (Table S6 in Supplementary Material).

Before treatment, a positive correlation between IFN-γ-producing CD4 cells and TEM CD4 cells was found, which suggested that IFN-γ production in CD4 lymphocytes was mainly performed by TEM cells. Interestingly, we found that plasmablasts were positively correlated to NK bright cells and negatively to NK dim cells in MS patients before treatment. After 6 months of fingolimod therapy, multiple correlations between lymphocyte subpopulations were found due to the impact of fingolimod on the relative percentages of lymphocytes. Helper T cells were positively correlated to memory and regulatory B cells and negatively correlated to naïve B cells and NK cells. Similarly, cytotoxic T cells were positively correlated to NKT cells and negatively to NK cells. The correlation matrices are shown in Figure S2 in Supplementary Material. All data for lymphocyte subpopulations are shown in published repository (26).

### Selected Lymphocyte Subpopulations Could Predict the Treatment Response to Fingolimod

**Figure 2:**
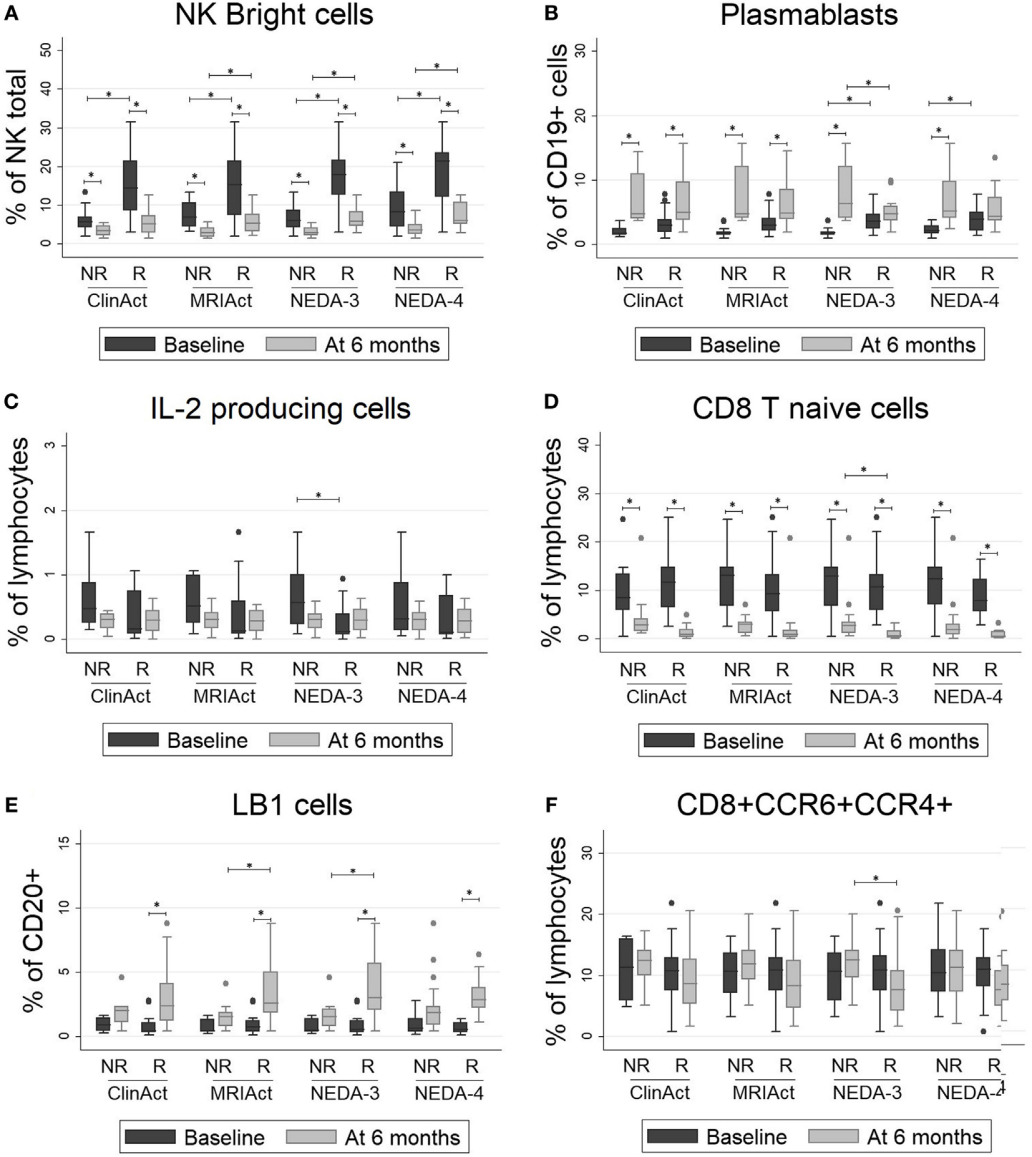
(**A–F**) Lymphocyte subpopulations with significant differences between responder (R) and non-responder (NR) patients: NK bright cells, plasmablasts, IL2-producing cells, CD8 T naïve cells, LB1 cells and CD8+CCR6+CCR4+ cells. The percentages of lymphocyte subpopulations at baseline and at 6 months of treatment are shown for different subgroups according to clinical response by four different measure ways: ClinAct: clinical activity (NR = clinical activity present, R = clinical activity absent); MRIAct: radiological activity (NR = radiological activity present, R = radiological activity absent); NEDA-3 (NR = no evidence of disease activity by NEDA-3 criteria, R = evidence of disease activity by NEDA-3 criteria); and NEDA-4 (NR = no evidence of disease activity by NEDA-4 criteria, R = evidence of disease activity by NEDA-4 criteria). The *p*-values were calculated using the Wilcoxon signed-rank test to compare differences before and after 6 months of therapy and using Mann–Whitney test to compare differences between subgroups. *p* < 0.001 was considered statistically significant after Bonferroni’s correction for multiple tests.

**Figure 3:**
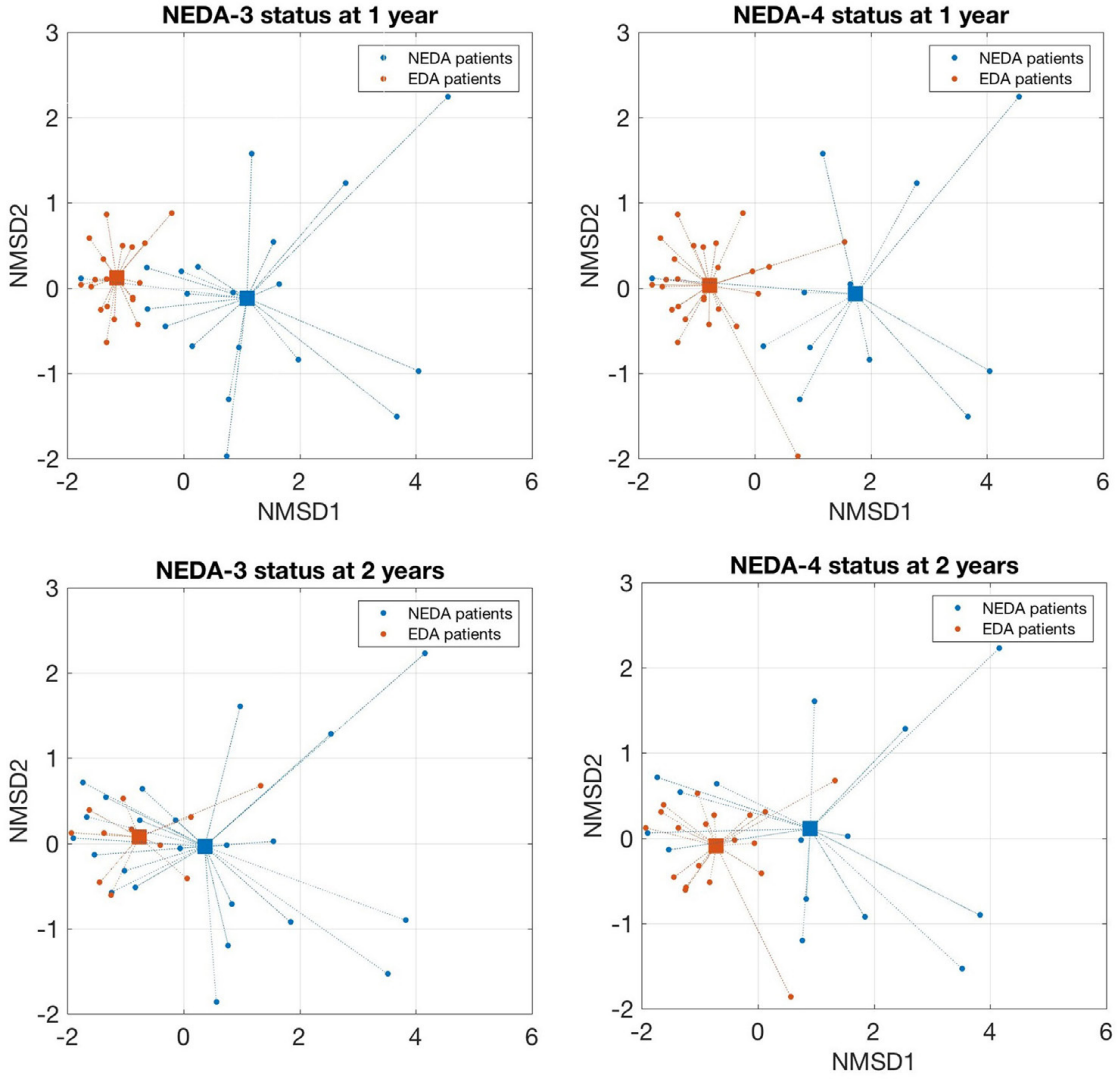
Nonparametric multidimensional scaling of selected lymphocytes subpopulations that discriminates responders and non-responder patients. A 2D representation by nonparametric multidimensional scaling (Seuclidean method) of the percentages of four selected lymphocyte subpopulations measured before treatment [natural killer (NK) bright, NK dim, plasmablasts, and IL-2-producing cells]. Individual patients and distance from the average of each condition are shown. Patients with a good response to fingolimod at 1 year (NEDA-3 and NEDA-4) were located in a well-differentiated region of space when the subpopulations are measured before treatment. At 2 years, this differentiation was less evident.

As shown in Figure 2, patients who achieved NEDA-3 and NEDA-4 status at 1 year had a significantly higher percentage of NK bright and plasmablast cells and a lower proportion of NK dim and IL-2-producing cells at baseline than NR patients (*p* < 0.001 for all). These differences were maintained after 6 months of treatment for NK bright cells. These patients were significantly more resistant to decreases in percentages of NK bright and LB1 cells and showed a greater decline of CD8 naïve T and CD8+ CCR4+ CCR6+ cells than NR patients after 6 months of treatment (*p* < 0.001 for all). These differences were maintained at 2 years, but they did not achieve statistical significance. The chemokine receptors CCR4 and CCR6 have been identified as markers of human Th17 cells that secrete IL-17 (27). We also found that the frequency of Th17 cells measured by surface markers was higher than their frequency estimated by intracellular staining, but this phenomenon could not be correlated to any of the response parameters. Logistic regression analysis showed a greater number of subpopulations with high regression coefficients (positive or negative) in pretreatment samples than samples obtained at 6 months. In pretreatment sample, the subpopulations that can be used as biomarkers of response to fingolimod for most of the target variables (clinical activity, radiological activity, NEDA-3, and NEDA-4) at 1 and 2 years are NK bright, NK dim, plasmablasts, and IL-2 producing cells. On the contrary, in the sample obtained at 6 months, the regression coefficients only highlight the naive CD8 T population as a possible predictor of response as is shown in Figure S3 in Supplementary Material. According to the values of these four baseline candidates for prediction, each patient was placed in a 2D representation by nonparametric multidimensional scaling, and we found that responder patients at 1 year were located in a well-differentiated region of space, as shown in Figure 3. At 2 years, the differentiation was less evident.

### Alteration of the Gene Expression Profile by Treatment

**Figure 4:**
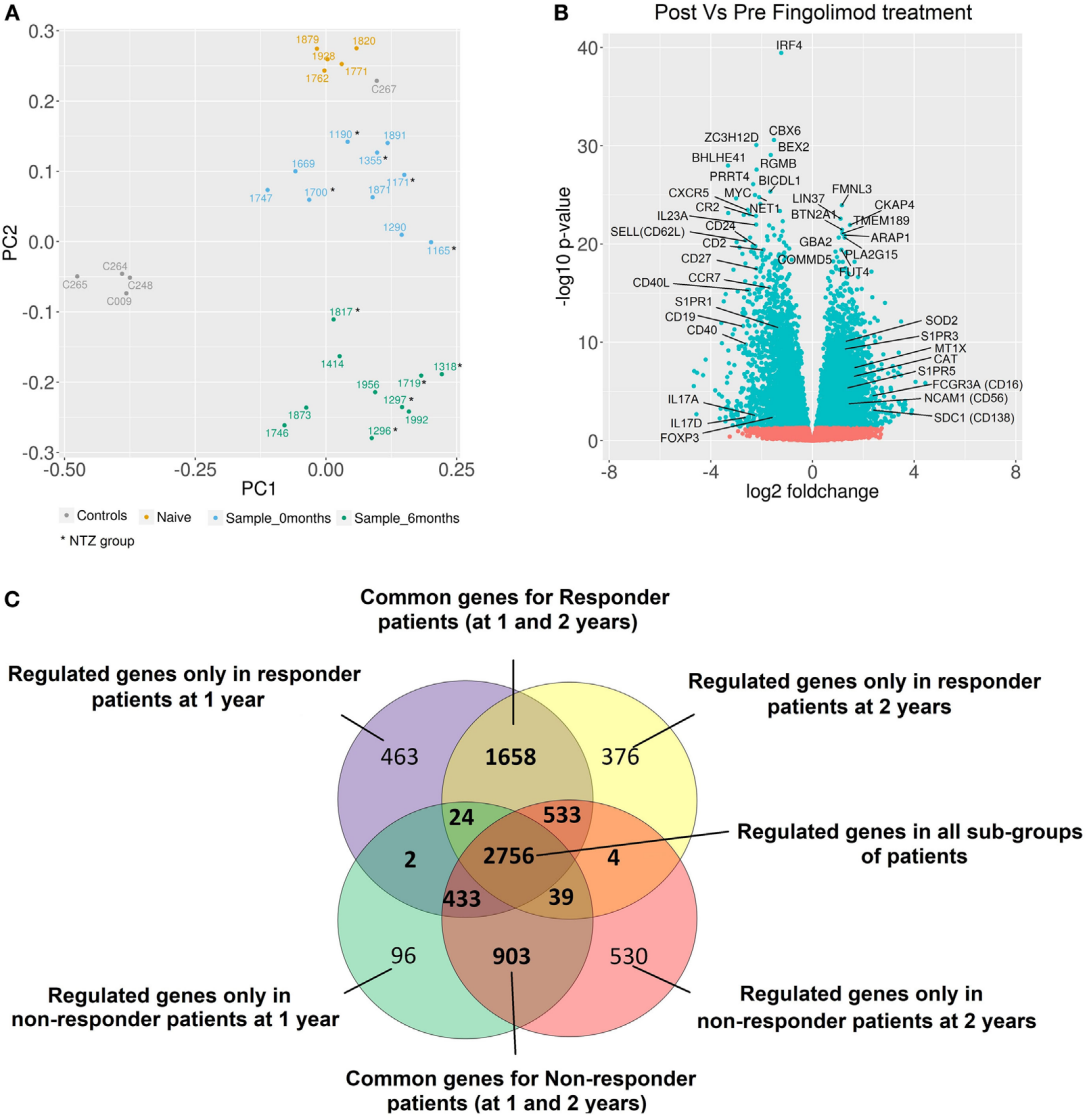
Alteration of gene expression profiles by fingolimod in peripheral blood mononuclear cells from multiple sclerosis (MS) patients. **(A)** The principal component analysis of gene expression profiles of all samples is shown. All healthy controls (gray) were transcriptomically similar except one of them (C267) who exhibited a gene expression profile more similar to naïve patient gene expression. This sample was not considered for further analysis. According to gene expression profiles, the samples from naïve patients (yellow) were located closer to samples from patients before treatment. The samples from patients after treatment (green) were located in a well-differentiated region of space indicating the strong fingolimod effect on gene expression. In samples from patients before (blue) and after treatment (green), no differences between NTZ and no-NTZ groups were found. **(B)** Volcano plot of differentially expressed genes (DEGs) between samples before and at 6 months of treatment for all samples. The most important genes related to MS and autoimmunity are indicated. **(C)** The Venn diagram shows the overlap of DEGs between different subgroups: responder and non-responder patients at 1 and 2 years. Responder patients = NEDA-4 patients and non-responder patients = EDA-4 patients (adjusted *p*-value < 0.05).

A principal component analysis of gene expression profiles of all samples is shown in Figure 4A. Naïve patients and HC were transcriptomically different from patients in our study. No transcriptional differences were found between the NTZ and no-NTZ group before treatment, which allowed the analysis of all patients without taking into account the previous treatment. Fingolimod exerts powerful transcriptional effects on PBMCs of MS patients. A total of 16,818 filtered probes were used for the differential analyses, resulting in the identification of 3,805 upregulated and 3,741 downregulated genes in response to fingolimod. As expected, genes related to mechanisms of action of fingolimod such as *S1PR1, SELL (CD62L)*, and *CCR7*, as well as genes implicated in sphingolipid metabolism *SPHK1* and *SHPK2* were downregulated after treatment. As shown in Figure 4B, fingolimod produced an anti-inflammatory effect by downregulating *CD40L, CD40, IRF4, CR2, IL-23A, CXCR5, CD24, CD2, CD27*, and *CD19*, as well as an increase in the expression of the anti-inflammatory cytokine genes and their receptors (*IL-10, IL10RA, IL10RB*, and *IL-15*) and the TNFRSF1A receptor gene, a regulator of inflammation. We also found an upregulation of antioxidant genes implicated in the control of reactive oxygen species (ROS), such as *SOD2, CAT, MT1X, MGST1*, and *MAOA*. All data for the expression levels, fold changes, and statistical significance in the DEG analysis are shown in published repository (26) and all data of RNA-seq are included in SRA database SRP132699 (28).

### Differential Modulation of the Gene Expression Profiles in Responder and NR Patients

After stratification based on drug response, we observed that the number of DEGs was higher in responder than NR patients at 1 and 2 years (~2,500 genes vs ~1,500 genes), indicating that the regulation of gene transcription is intimately linked to the treatment response. *C3* and *IFIT1* genes were upregulated, and genes such as *FOXP3* and *IL17A* were downregulated in response to fingolimod only in responder patients. By contrast, *EGR4* and *MAPK11* genes were downregulated exclusively in NR patients (Figure 4C).

In the S1P pathway, we found that fingolimod inhibited antiapoptotic processes through *AKT* and *NOS3* downregulation and could play a role in decreasing stress-fiber formation and T cell maturation through *NFKB* downregulation. We did not observe significant differences between responder and NR patients in the S1P pathway except for an upregulation in responders and a downregulation in NRs of *MAPK3* (at 1 and 2 years). Similarly, in the oxidative stress response pathway (Ontology terms: PW0000378), we observed a downregulation of *FOS* and an upregulation of *HMOX1* in patients with evidence of disease activity (EDA) at 1 and 2 years (Figure S4 in Supplementary Material).

### Baseline Signature in Responder Patients

**Figure 5:**
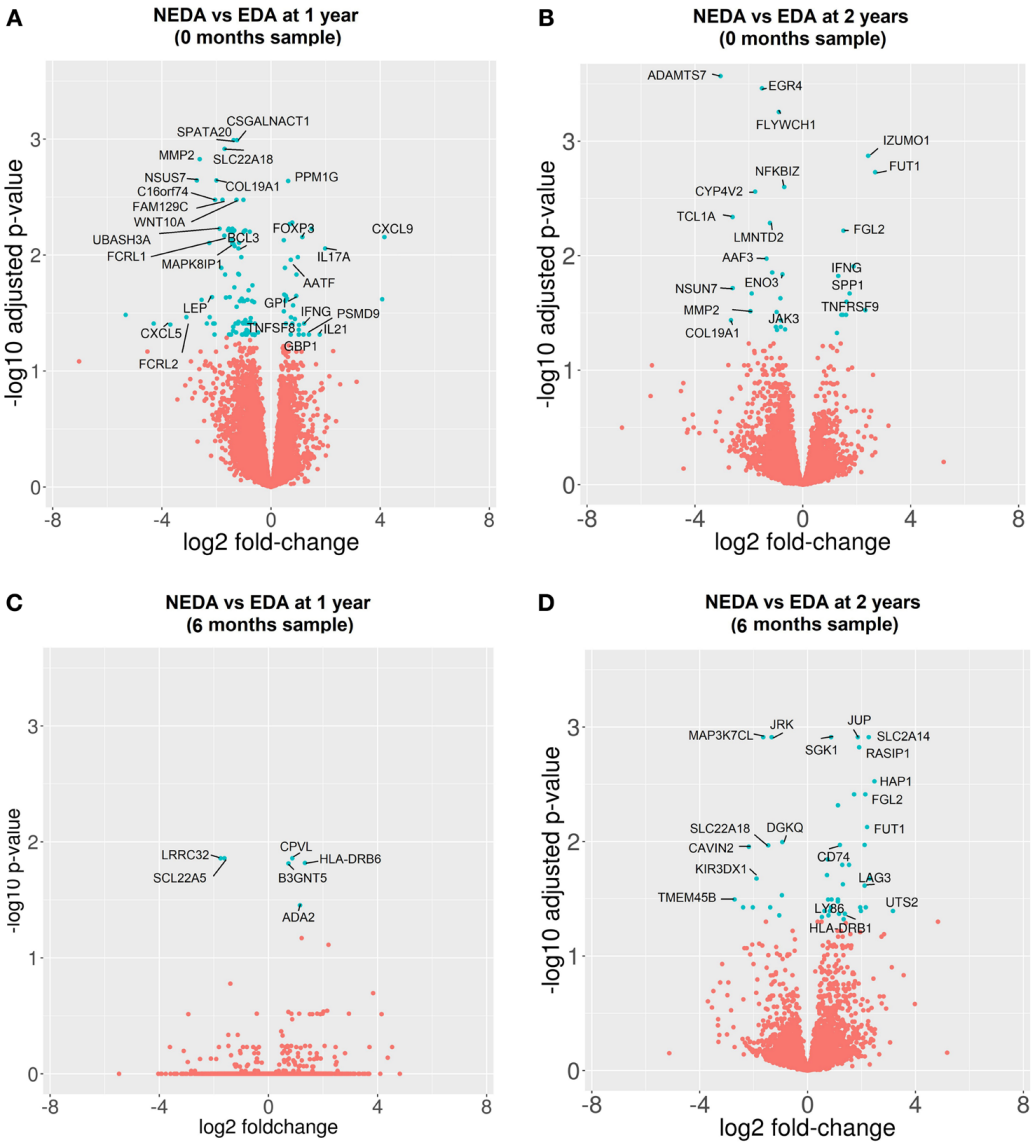
Differentially expressed genes (DEGs) between responder (R) and non-responder (NR) patients before and after 6 months of treatment. Volcano plots of DEGs between R and NR patients. **(A,B)** Samples obtained before treatment. Clinical response measured at 1 year **(A)** and 2 years **(B)**. **(C,D)** Samples obtained after 6 months of treatment. Clinical response at 1 year **(C)** and 2 years **(D)**.

The gene expression profile before treatment was compared among responder and NR patients. Before treatment, 127 genes were differentially expressed when the response was measured at 1 year and 36 genes at 2 years (Figures 5A,B). In responder patients, overexpression of genes related to cytokine secretion (*IL-21, IFNG, IL-17A*, and *CXCL9*), activation of the T cell response (*TNFSF8* and *TNFRS9*), activation of regulatory mechanisms (*FOXP3* and *SPP1*), immunoglobulin secretion (*GPI*), apoptosis (*AATF*) and processing of class I MHC (*PSMD9*), as well as a down-expression of genes related to modulation of the innate immune response (*LEP* and *B3GAT1*), regulation of the Th1 response (*UBASH3A* and *MAPK8IP1*), immunoglobulin receptors (*FCRL1, FCRL2*, and *JAK3*), and B lymphocyte response (*BCL3*) before treatment was observed. In addition, 10 DEGs selected for validation by qPCR showed consistent expression between the qPCR and RNA-seq results.

When the gene expression profile was compared at 6 months between subgroups to determine the differential effect of fingolimod according to the clinical response, we found 6 DEGs in responder vs NR patients at 1 year and 51 DEGs at 2 years. The good responders showed a downregulation of specific markers of activated Tregs such as *LRRC32*, an upregulation of the cellular growth factor *ADA-2*, apoptotic genes such as *SGK1* and *BCL2L13* and genes related to the antigenic presentation by MHC-II such as *CD74, HLA-DRB6, HLA-DRA*, and *HLA-DRB1*. The most representative DEGs are shown in Figures 5C,D. A summary of the expression levels of the most representative genes and PCR validation are shown in Table S7 in Supplementary Material.

### GSEA of DEGs

**Figure 6:**
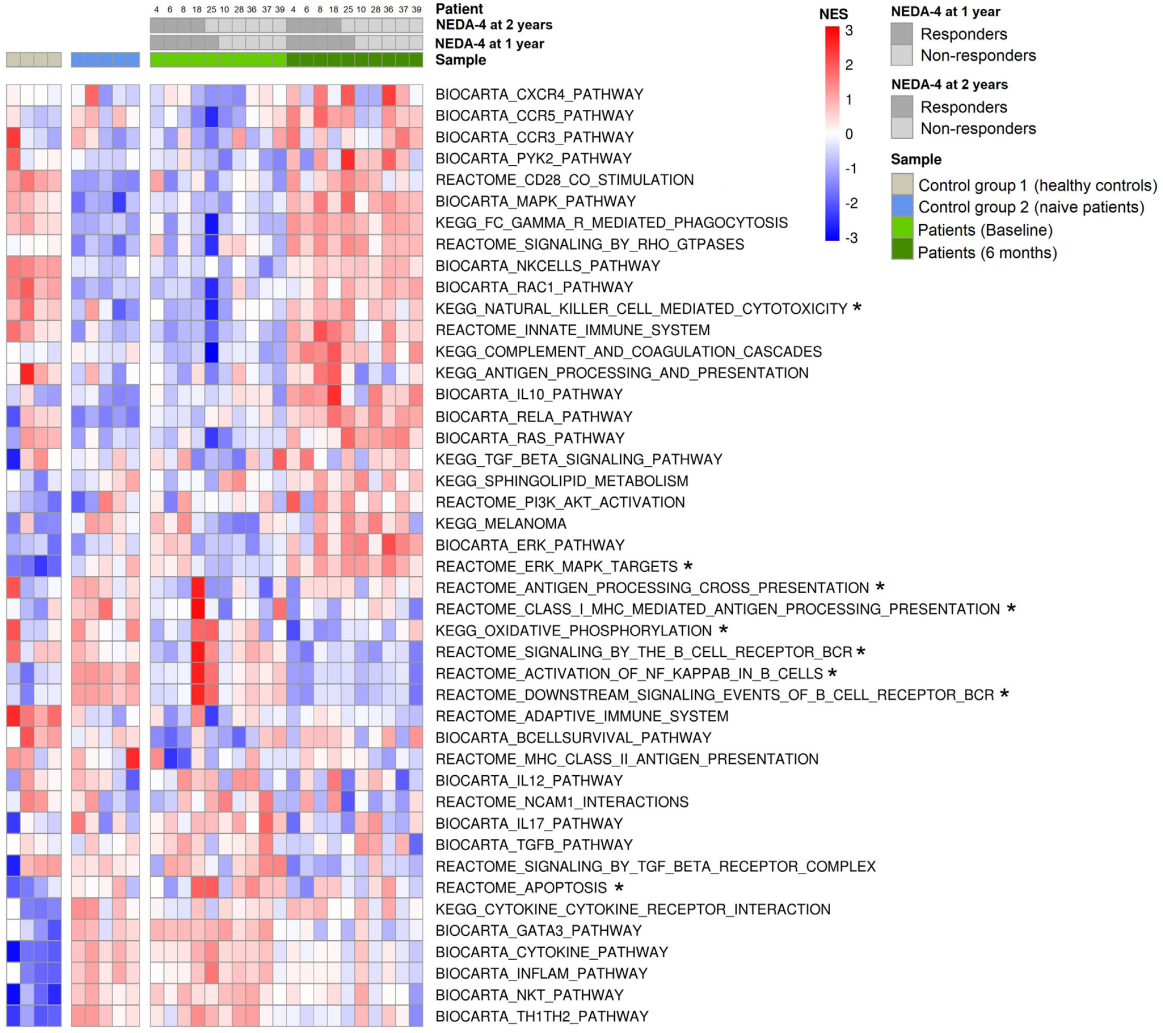
Gene set enrichment in response to fingolimod. Heatmap of significantly enriched pathways related to multiple sclerosis, autoimmunity, and fingolimod mechanisms of action that achieved significant enrichment in gene set enrichment analysis (GSEA) [false discovery rate (FDR) *q*-values <0.25]. GSEA was performed using GSEA software v2.2.1 obtained from the Broad Institute, and the molecular signature database of gene sets used were Biocarta, KEGG, and Reactome. Normalized enrichment scores (NES) are indicated by the color scale, with red representing positive enrichment and blue indicating negative enrichment compared with the median NES for each pathway across all donors. Color key annotation indicating the source of each sample (healthy controls, naïve patients, patients at baseline, and patients at 6 months of fingolimod treatment) and NEDA-4 status at 1 and 2 years (responder: NEDA-4, non-responder: EDA-4) for the 10 patients (4, 6, 8, 18, 25, 10, 28, 36, 37, and 39). *Gene sets enriched before treatment only in responder patients at 1 and 2 years.

The most important genes related to immunity and MS that achieved significant enrichment (positive or negative) after treatment are shown in Figure 6. Fingolimod was observed to exert an important transcriptional effect on PBMCs of MS patients, approaching a profile more similar to the HC profile. The gene sets containing genes related to S1P signaling such as MAPK/ERK, Rho kinases, RAC1, Ras, and sphingolipid metabolism, as well as the genes participating in IL-10 production and innate immune activation such as NK cells, Fc-mediated phagocytosis, CCR3, CCR5, CXCR4, the complement cascade, the innate immune system, and apoptosis were positively enriched in response to fingolimod. In our study, fingolimod downregulated gene sets related to cytokine production, such as the IL12 pathway, IL-17 pathway, and cytokine pathway, and of gene sets implicated in Th1, Th2, and Th17 response, such as Th1/Th2 pathway and GATA3. After stratification based on response to the drug, we observed that antigen processing cross-presentation and oxidative phosphorylation pathways before treatment were positively enriched exclusively in responder patients.

The normalized enrichment scores, *p*-values, and FDRs of significantly enriched gene sets implicated in the immune response and MS are summarized in Table S8 in Supplementary Material.

### Predictive Model of the Fingolimod Response

From the baseline cytometry and RNA-seq variables that showed statistically significant differences between responder and NR patients, we selected two lymphocyte subpopulations (NK bright and plasmablasts) and three genes (*FOXP3, FCRL1*, and *GPI*).

**Figure 7:**
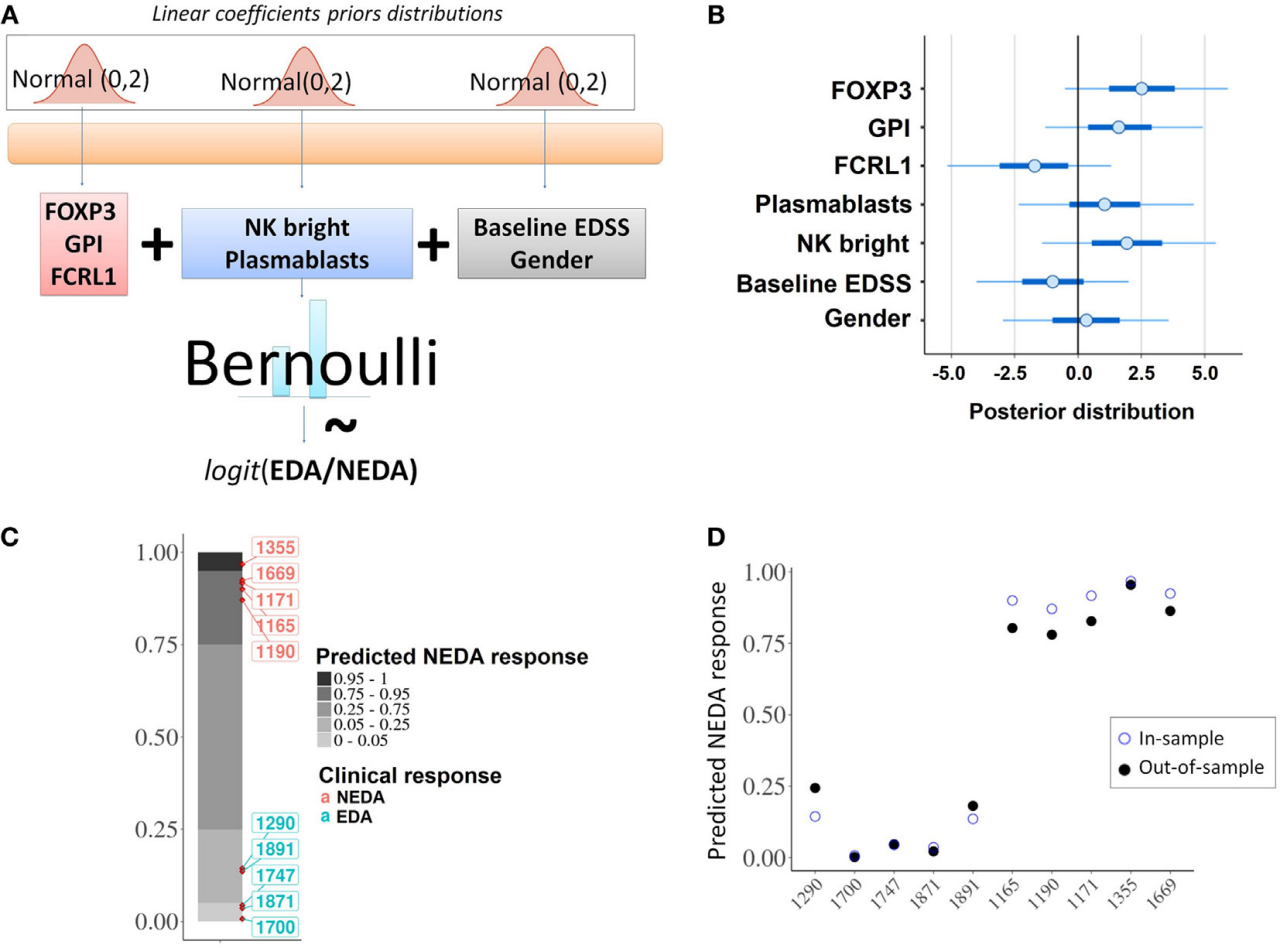
Predictive model. **(A)** Schematic representation of the probabilistic linear model for the fingolimod response. **(B)** Individual contributions of each of the selected predictors; the graph shows the posterior distribution of the estimated coefficient for the linear model, indicating the mean (dots), 50% quantile (thick line) and 95% quantile (thin line). **(C)** In-sample posterior predictive check; the model was correctly fitted to the given data predicting the expected response for the 10 considered patients. **(D)** In-sample vs out-of-sample prediction comparison; out-of-sample predictions using an approximation of leave-one-out cross-validation (filled dots) were compared with in-sample predictions (empty dots).

With these five variables the model was built (Figure 7A) and the individual contribution of each variable to the fitted model was evaluated (Figure 7B). An in-sample posterior prediction check indicated that the model was correctly fitted to the provided data (Figure 7C). The exact leave-one-out cross-validation showed that the model could be expected to perform well for out-of-sample predictions (Figure 7D).

### Validation of Predictive Model

The differential expression of *FOXP3, FCRL1*, and *GPI* between responder and NR was validated by qPCR in the remaining 30 patients who were not used for the analysis of RNA-seq and the results are shown in the Table 2.

**Table 2:** Quantitative PCR (qPCR) validation of differentially expressed genes in RNA-seq results.

	Discovery					Validation			
	Next-generation RNA sequencing (RNA-seq) *n* = 10					qPCR *n* = 30			
	Mean of expression levels		log2 FC^ 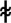 ^	*p*-Value*	*p*-Value adj^~^	Relative expression ratio^†^		FC^¶^	*p*-Value^‡^

Gene	Responder (*n* = 4)	Non-responder (NR) (*n* = 6)				Responder (*n* = 8)	NR (*n* = 21)		
FOXP3	3.26 ± 0.42	1.67 ± 0.49	1.14	1.48E−05	0.007	0.03 ± 0.04	0.22 ± 0.22	7.3	0.005
GPI	25.6 ± 2.78	18.4 ± 0.84	0.47	0.00016	0.03	2.21 ± 1.4	10.31 ± 10.51	4.6	0.012
FCRL1	0.74 ± 0.31	2.79 ± 1.33	−1.88	7.78E−06	0.005	0.69 ± 0.81	0.14 ± 0.14	−4.9	0.024

*In samples collected before treatment, the gene expression levels measured by RNA-seq of four responder vs six NR patients (at 2 years) were compared and three genes were selected for predictive model since they were differentially expressed between responder and NRs. The differential expression of these three genes (FOXP3, GPI, and FCRL1) were validated by qPCR finding statistically significant differences between responder and NRs patients for the three genes*.

*^
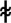
^The log2 (fold-change) or log2FC is the log-ratio of a gene expression values in two different biological conditions, in this case responder vs NR*.

*The estimated significance level (p-value) of differential gene expression was calculated with DESeq2, which uses a negative binomial distribution.

*^~^p-Values were corrected to account for multiple hypotheses testing using Benjamini and Hochberg false discovery rate (FDR) adjustment. Genes with an FDR less than or equal to 0.05 were selected as differentially expressed*.

*^†^Relative expression of the target genes vs three reference genes using the 2^−ΔΔCt^ method*.

*^¶^Fold change is the ratio of the gene expression values*.

*^‡^p-Value was calculated using the t-test to compare means of expression levels*.

**Figure 8:**
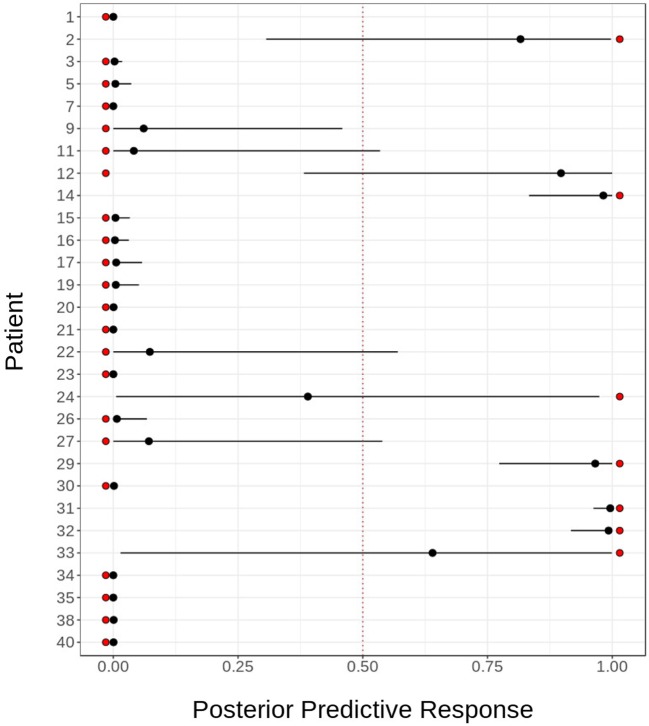
Sensitivity and specificity of predictive model. Predictive model validation: in order to assess the goodness of our predictive model for fingolimod response, we validated it using cytometry and transcriptomic data for 29 patients. The figure shows the actual response (red dots) against the posterior distribution of the predicted response, represented by its mean (black dot) and 95% quantile (black line). The model fails to predict the response for two patients: 1,315 and 1,655.

The model was tested in this cohort for the good response prediction with a sensitivity of 85%, a specificity of 95%, a positive predictive value of 85%, and negative predictive value of 95%. The mean squared error is 0.1111438. The response probability obtained for each sample and the comparison with real clinical response is shown in Figure 8. The mean, lower, and upper posterior predictive for each sample in the validation was summarized in Table S9 in Supplementary Material.

## Discussion

Multiple sclerosis patients treated with fingolimod exhibit lymphopenia with a decrease in TN, TCM, memory B, and NK bright cells and a relative increase in TEM, TEMRA, naïve B, transitional B, plasmablast, NK, and NKT cells in peripheral blood (29–33).

In a previous report, pretreatment differences were found between the responder and NR patients in the transitional B and RTE cells (34, 35). Our study differed from those results probably due to differences in patient selection. In our study, naïve patients were excluded and the percentage of patients whose previous treatment was NTZ was 45% while the previous report included 37.5% of naive patients and only 20.2% of patients whose previous treatment was NTZ (*n* = 7). In our study, the percentage of transitional B cells in the pretreatment samples was higher in NTZ group. That is consistent with the increase in transitional B cells and RTEs induced by NTZ that can remain even months after the withdrawal (36–38). For the analysis of RTE and transitional B cells as cytometry biomarkers, we think that the stratification of the patients according to the previous treatment is necessary and patients who have received NTZ during the last months should be excluded. In addition, although we did not analyze the RTE cells by cytometry, we did not find differences in the expression levels of the genes coding for their surface markers CCR7, CD45RA, CD31, and PTK7 (*CCR7, PTPRC, PECAM1*, and *PTK7*) between responder and NR patients before treatment.

Preliminary studies showed that *CD58, CX3CR1, CCR1, CCR2, HLA-DRA*, and *HLADRB1* genes were upregulated in CD4 cells and *IFNG, IFNGR1, FASLG, IL-2RB, GZMA*, and *MAPK1* genes were upregulated in CD8 cells after 3 months of fingolimod treatment. In addition, the expression of *CCR7, TNFSF8, CD27*, and *LEF1* was downregulated after treatment (39, 40), but a correlation with clinical response was not assessed.

In this study, as in most studies of this type (41–44), the PBMCs are considered as a valid cellular group since they are able to reflect patterns of expression characteristic of certain diseases and treatment effects. The majority of studies evaluating the transcriptomic effect of a treatment do it on whole PBMCs. In fact, the changes in expression levels on PBMCs in patients with MS treated with IFN-β are known as IFN signature and it has been proposed as a response marker (44). In addition, a recent study showed that the transcriptional change induced by fingolimod belong almost entirely to CD4 lymphocytes and based on these results, the efficiency of the sorting could be lower than the analysis of the entire population of PBMCs (45). Although in our study a discrimination by lymphocyte subtypes was not conducted, we obtained the same results as the previously described genes when the effect of fingolimod was analyzed.

It is clear that fingolimod exerts a potent anti-inflammatory effect by decreasing the percentage of cytokine-producer T and B cells (7). In this study, we confirmed this effect not only at the cellular level but also at the molecular level, since fingolimod induced a downregulation of genes such as *CD40L, CD40, IRF4, CR2, IL-23A, CD2, IL17A*, and *IL17D*. Similarly, the cytokine activity pathways were downregulated after treatment.

Natural killer cells are lymphocytes of the innate immune system that are involved in defense against viruses, bacteria, or parasites, or malignant transformation. NK bright and invariant NKT cells, a subset of NKT, are often related to regulatory processes (46). In MS patients, a high proportion of NK bright cells has recently been associated with stable MRI (47), and we found that a higher percentage of these cells before treatment was associated with a good response and that they decreased to a lesser degree after treatment in responder compared with NR patients. In our study, fingolimod not only increased the percentage of NK cells but it also upregulated *NCAM1* (CD56) and *FCGR3A* (CD16a) at the transcriptional level; an enrichment of the NK and innate response pathways was also observed as a result of the therapy. The role of innate immunity in MS has recently been appreciated: its cells are capable of producing direct damage to CNS myelin and play an important role as a source of ROS contributing to axonal damage (48). Despite this phenomenon, in our study, fingolimod downregulated genes implicated in oxidative stress and inhibition of stress-fiber formation through the *RhoA–ROCK1–NFKB* pathway, suggesting that the antioxidant effect of fingolimod was not dependent on innate immune activation. *In vitro*, fingolimod exerts an antioxidant effect, and in the EAE model, it blocks astrocyte activation and nitric oxide production (49). In MS patients, lower serum levels of ROS were observed in those treated with fingolimod compared with the first line-treated patients, and a correlation between ROS levels and disease duration was also observed (50).

The percentage of CD4 T regulatory cells do not differ between patients and controls, but their function is compromised (maturation and migration) (51) and the transcription factor FoxP3, crucial for the function of regulatory T-cells, has been reported to be diminished in RRMS patients (52). According to previous reports (7), we found that fingolimod induces an increase in the percentage of regulatory T lymphocytes but surprisingly a downregulation of expression levels of *FOXP3* was observed. This divergence had already been previously described and commented on in a study by van Pesch et al. (53) and it could be partially explained due to the reduction of the absolute number of Tregs. FoxP3 is not only a transcription factor of Tregs but it is also expressed transiently in effector T cells after its activation (54). The decrease in lymphocyte activation in response to fingolimod could contribute to the decrease in FOXP3 expression levels. On the other hand, the increase in the expression of IL-10, IL10RA, IL10RB, IL-15, and TNFRSF1A reveals the powerful inductive effect of regulatory mechanisms in response to fingolimod observed in this study.

In the present study, we found that higher levels of *FOXP3* before treatment were correlated with a good response, raising the possibility that patients with stronger regulatory mechanisms can obtain a greater benefit from immunomodulatory therapies.

*FOXP3* and *LRRC32*, a recently identified specific marker of activated regulatory T-cells (55), were downregulated by fingolimod only in responder patient as well as the ADA2 gene, also known as CECR1, which intervenes in the activation of Tregs through binding to the CD39 receptor (56). That suggested that once the excessive inflammatory response is controlled the compensatory mechanisms are less necessary.

Upregulation of the apoptotic pathway indicated that fingolimod-induced programmed cell death, a mechanism that might contribute to lymphopenia. However, we observed an overexpression of *ADA2, SGK1*, and *BCL2L13* after 6 months of treatment only in responder patients, potentially suggesting a differential effect of fingolimod on the mechanisms of cell proliferation and differentiation.

B cells and B cell activation are recognized to play a key role in MS pathophysiology (57). The tumor necrosis factor receptor superfamily member 13B (TNFSF13) and its ligand B-cell activating factor have also been shown to play an important role in the proliferation and differentiation of B cells in MS. In fact, oligoclonal bands have been associated with increased levels of TNFSF13B in the CSF of MS patients (58). The CXCL13 and CXCR5 molecules have also been related to the recruitment of B cells to the CNS during neuroinflammation (59). We observed an important downregulation of *IRF4* (a highly expressed molecule in activated B cells) (60), *CD27* (a memory B cell marker) and *CXCR5*, as well as a decrease in the percentages of memory B cells after treatment. Interestingly, we observed a higher percentage of plasmablasts and an overexpression of *CXCL13* before treatment in patients who achieved a NEDA response, suggesting that the modulation of B cell activation plays an important role in the clinical response to fingolimod. The plasmablasts are cells highly differentiated toward the production of antibodies and are related to clinical and radiological activity in EM (61). In this study, there was a higher percentage of plasmablasts at baseline in responder patients to fingolimod, as well as higher transcriptional activity in terms of antibody synthesis. An example of this observation is the basal overexpression of the GPI gene that codes for glucose phosphate isomerase (GPI), a lymphokine that induces the production of immunoglobulins (62). In rheumatoid arthritis, GPI levels correlate with disease activity and it has been shown that fingolimod can prevent GPI-induced outbreaks (63). An opposite behavior was observed in this study in the genes that code for the immunoglobulin receptors FCRL1 and FCRL2 indicating that the responder patients despite exhibiting an increase in the production of immunoglobulins have a lower activity of their receptors. On the other hand, low levels of FCRL1 and 2 have been associated with an increase in radiological activity in MS (64) and this is in agreement with the fact that fingolimod exerts a better effect in patients with a more inflammatory component.

The interleukin-2 receptor α-subunit (IL-2RA) has generated great interest since polymorphisms of this gene have been associated with the risk of developing MS (65) and due to the recent approval of daclizumab, a humanized IgG1 monoclonal antibody directed against the alpha subunit of the high-affinity IL-2 receptor, which has demonstrated high efficacy in the control of MS activity (66). Fingolimod treatment decreases soluble IL-2RA plasma levels (67). In accordance with those results, we detected a decrease in IL-2-producing cells and a downregulation of the IL-2RA gene in PBMCs of treated MS patients. Although we observed a differential decrease in IL-2-producing cells between NEDA and EDA patients, no differences were found at the transcriptional level.

Surprisingly, we found no differences in the transcriptomic profile of PBMCs from patients of NTZ-group and no-NTZ group although previous reports of transcriptional changes induced by NTZ and IFN-β in MS patients have been published (68, 69). In the NTZ-group, this could be explained by the absence of long-lasting immunological and transcriptional changes by NTZ after the washout period and we could think that the immune reactivation is related just to the reversibility of the effect of NTZ on the lymphocyte transmigration and not to activation changes. In the no-NTZ group, the absence of an interferon/glatiramer acetate signature could be related to the lack of effectiveness of first-line DMTs, which was the main reason for the switch to NTZ.

In addition to the differential effect of fingolimod between patients, the baseline cellular and genetic characteristics can help us to predict the clinical response to the drug. High percentages of NK bright cells and plasmablasts, high expression levels of *FOXP3* and *GPI*, as well as low expression levels of *FCRL1* and low EDSS before treatment could be correlated with a good response, which allowed us to design a prediction model of the fingolimod response that must be validated in an independent cohort.

## Conclusion

Fingolimod treatment affects practically all lymphocyte subpopulations and exerts a strong effect on genetic transcription. In PBMCs from MS patients, fingolimod induces a decrease in the inflammatory response at cellular and molecular levels, improves regulatory mechanisms, and has important antioxidant effects. The differential fingolimod-induced effects on PBMCs at 6 months as well as the baseline cellular and molecular characteristics can be used to predict the clinical response at 1 and 2 years. We postulate that a combination of cellular (NK bright and plasmablasts), molecular (*FOXP3, GPI*, and FCRL1), and clinical markers (EDSS and gender) are possible response biomarkers. However, our data are only descriptive, and more studies with a greater number of patients are necessary to validate this model.

## Ethics Statement

This study was carried out in accordance with the recommendations of Spanish Real Decreto 223/2004. The protocol was approved by the Clinical Research Ethics Committee of the Puerta de Hierro Hospital (CEIC). All subjects gave written informed consent in accordance with the Declaration of Helsinki.

## Author Contributions

IT, AL, and AG-M designed and conceived the study; IT performed most of the experiments; IT, AG, and CG performed cytometry measurements and analyzed the data; LR, RH, and AS prepared the samples for cytometry and RNA-seq; ER prepared the samples and performed qPCR analysis; LE performed measures of BVL by SIENA method; VE and MM performed the statistical analysis; MM, FA-S, and CF-T analyzed the transcriptomic data; IT wrote the paper. All authors revised and approved the submitted version.

## Conflict of Interest Statement

AG-M has received honoraria for lecturing, consulting, or travel expenses from Bayer, Biogen-Idec, Merck, Teva, Novartis, Roche, Almirall, and Genzyme, and research grants from Merck and Novartis. IT has received honoraria for lecturing and travel expenses from Merck, Teva, Novartis, and Genzyme. LE has received honoraria for lecturing and travel expenses from Novartis. The other authors do not have any conflicts of interest to report.

## Abbreviations

ARR, annualized relapse rate; BAFF, B-cell activating factor; BVL, annual brain volume loss; CDP, confirmed disability progression; CNS, central nervous system; DEGs, differentially expressed genes; DMTs, disease-modifying therapies; EDA, evidence of disease activity; EDSS, Expanded Disability Status Scale; FCAs, fluorochrome-conjugated antibodies; FDR, false discovery rate; GdE, gadolinium-enhanced T1 lesions; *GSEA*, gene set enrichment analysis; HC, healthy controls; IL-2RA, interleukin-2 receptor α-subunit; MRI, magnetic resonance imaging; MS, Multiple sclerosis; NEDA, no evidence of disease activity; NES, normalized enrichment scores; NGS, next-generation sequencing; NK, natural killer; NR, non-responder; NTZ, natalizumab; PCA, principal component analysis; PBMCs, peripheral blood mononuclear cells; qPCR, quantitative PCR; R, responder patients; ROS, reactive oxygen species; RRMS, relapsing–remitting multiple sclerosis; S1P, sphingosine-1-phosphate; T2w, T2-weighted lesions; TCM, central memory; TEM, effector memory T cell; TEMRA, terminally differentiated effector T cell; TN, T naïve cells; TNFSF13, tumor necrosis factor receptor superfamily member 13B; Tregs, regulatory T cells.
