# Supplementary material for: Immunophenotype and Transcriptome Profile of Patients With Multiple Sclerosis Treated With Fingolimod: Setting Up a Model for Prediction of Response in a 2-Year Translational Study
Source: Front Immunol. 2018 Jul 25;9:1693. doi: 10.3389/fimmu.2018.01693 (PMC6068231; doi:10.3389/fimmu.2018.01693)

Supplementary Material

Immunophenotype and Transcriptome Profile of Patients with Multiple Sclerosis Treated with Fingolimod. Setting Up a Model for Prediction of Response in a 2-Year Translational Study.

Author names.

Irene Moreno Torres MD, Coral González García PhD, Marco Marconi PhD, Aranzazu García Grande PhD, Luis Rodríguez Esparragoza MD, Víctor Elvira PhD, Elvira Ramil PhD, Lucía Campos Ruíz, Ruth García Hernández MSC, Fátima Al-Shahrour PhD, Coral Fustero MSC, Alicia Sánchez Sanz MSC, *Antonio García Merino MD PhD, *Antonio José Sánchez López PhD.
* Both authors are senior scientist and co-corresponding on this work.

Correspondence.

Dr. Antonio García Merino
e-mail: jgarciam.hpth@salud.madrid.org.

Dr. Antonio José Sánchez López
e-mail: asanchezl.hpth@salud.madrid.org

Supplementary Table 1. Lymphocyte subpopulations analyzed by flow cytometry.

PANEL	ANTIBODIES	SUBPOPULATIONS ANALYZED
**1**	anti-CD3 FITC, anti-CD56 PE, anti-CD20 Per-CP vio 770, anti-CD4 PE vio 770 and anti-CD8 APC	**T Lymphocytes**: CD3^{+}CD20^{-}CD56^{-}<br>**Helper T cells**: CD3^{+}CD4^{+}CD20^{-}CD56^{-}<br>**Cytotoxic T cells**: CD3^{+}CD8^{+}CD20^{-}CD56^{-}<br>**B lymphocytes**: CD20^{+}CD3^{-} CD56^{-}<br>**Natural killer cells (NK)**: CD56^{+}CD20^{-}CD3^{-}, CD56^{dim}CD20^{-}CD3^{-} and CD56^{bright}CD20^{-}CD3^{-}<br>**Natural killer T cells (NKT)**: CD3^{+}CD56^{+}CD20^{-}
**2**	anti-CD45RA Vio green, anti-CD39 FITC, anti-CCR7 PE, anti-CD8 PerCP, anti-CD127 PE Vio 770, anti-CD25 APC and anti-CD4 APC vio 770	**Naive T cells (Tn)**: CD3^{+} CD4^{+} CCR7^{+} CD45RA^{+} and CD3^{+} CD8^{+} CCR7^{+} CD45RA^{+}<br>**Central memory T cells (TCM)**: CD3^{+} CD4^{+} CCR7^{+} CD45RA^{-} and CD3^{+} CD8^{+} CCR7^{+} CD45RA^{-}<br>**Effector memory T cells (TEM)**: CD3^{+} CD4^{+} CCR7^{-} CD45RA^{-} and CD3^{+} CD8^{+} CCR7^{-}CD45RA^{-}<br>**Effector memory RA T cells (TEMRA)**: CD3^{+} CD4^{+} CCR7^{-} CD45RA^{+} and CD3^{+} CD8^{+} CCR7^{-} CD45RA^{+}<br>**Regulatory T cells (Reg T)**: CD3^{+} CD4^{+} CD25^{high} CD127^{down}
**3**	anti-CD3 FITC, anti-CD27 PE, anti-CD20 Per-CP vio 770, anti-CD11b APC and anti-CD43 APC vio 770	**CD20 Naive B cells (Naïve B)**: CD20^{+} CD3^{-} CD27^{-} CD43^{-}<br>**CD20 Memory B cells (Mem B)**: CD20^{+} CD3^{-}CD27^{+} CD43^{-}<br>**B1 cells (B1)**: CD20^{+} CD3^{-}CD27^{+} CD43^{+}<br>**B1 CD11b+ cells**: CD20^{+} CD3^{-} CD27^{+} CD43^{+} CD11b^{+}
**4**	anti-CD24 FITC, anti-CD27 PE , anti-CD38 PE-vio 770, anti-IgM APCand anti-CD19 APC vio770	**CD19 Naive B cells (NaiveB)**: CD19^{+} CD27^{-} IgM^{+}<br>**CD19 Memory B cells (MemB)**: CD19^{+} CD27+<br>**Class switched memory B cells (CS MemB)**: CD19^{+} CD27^{+} IgM^{-}<br>**Non class switched memory B cells (NoCS MemB)**: CD19^{+} CD27^{+} IgM^{+}<br>**Immature B cells (Immat B)**: CD19^{+} CD27^{-} IgM^{-}<br>**Transitional B cells (Transit B)**: CD19^{+} CD27^{-}CD24^{high} CD38^{high}<br>**Plasmablasts (PB)**: CD19^{+} CD24^{-}CD38^{high} IgM^{-}<br>**Regulatory B cells (Reg B)**: CD19^{+} CD24high CD27^{+} and CD19^{+} CD24high CD27^{+}CD38int
**5**	anti-CD3 Vio green, anti-CD27 PE , anti-CD138 PE-vio 770, anti-CD5 APC and anti-CD19 APC vio770	**Plasmatic cells (PC)**: CD19^{+} CD3^{-}CD27^{+} CD138^{+}<br>**CD5+ B cells**: CD19^{+} CD5^{+}
**6**	Surface staining: anti-CD3 Vio green, anti-CD161 FITC, anti-CD8 PerCP, anti-CCR6 PE vio 770 and anti-CCR4 APC.<br>Intracellular staining: anti-IL17A PE.	Th17 cells by surface markers vs intracellular staining:<br>IL17^{+}, CD3^{+}CD8^{-} IL17^{+} and CD3^{+}CD8^{+} IL17^{+}<br>CD3^{+} CD8^{-}CCR6^{+} CCR4^{+} vs CD3^{+} CD8^{-}CCR6^{+} CCR4^{+} IL17^{+}<br>CD3^{+} CD8^{-}CD161^{+} CCR6^{+} CCR4^{+} vs CD3^{+} CD8^{-}CD161^{+} CCR6^{+} CCR4^{+} IL17^{+}<br>CD3^{+} CD8^{+}CCR6^{+} CCR4^{+} vs CD3^{+} CD8^{-}CCR6^{+} CCR4^{+} IL17^{+}<br>CD3^{+} CD8^{+}CD161^{+} CCR6^{+} CCR4^{+} vs CD3^{+} CD8^{-}CD161^{+} CCR6^{+} CCR4^{+} IL17^{+}
**7**	Surface staining: anti-CD3 Vio green and anti-CD8 PerCP,.<br>Intracellular staining: anti-IL17A PE, anti-IFNγ FITC and anti-IL2 APC vio770	IFNγ^{+}, CD3^{+} CD8^{-} IFNγ^{+} and CD3^{+} CD8^{+} IFNγ^{+}<br>IL2^{+}, CD3^{+} CD8^{-} IL2^{+} and CD3^{+} CD8^{+} IL2^{+}<br>IL17^{+} IFNγ^{+}, CD3^{+} CD8^{-} IL17^{+} IFNγ^{+} and CD3^{+} CD8^{+} IL17^{+} IFNγ^{+}

Supplementary Table 1. Lymphocyte subpopulations analyzed by flow cytometry.

Seven cytometry panels with a different combination of antibodies were designed to characterize 48 lymphocyte subpopulations according surface and intracellular markers. Prior to staining, all cells of each panel were pre-incubated with Fc-blocking reagent from Miltenyi Biotec. To exclude dead cells, 4′,6-diamidine-2′-phenylindole dihydrochloride (DAPI) was added to the surface staining panels (1-5) and the fluorescent reactive dye of LIVE/DEAD™ Fixable Dead Cell Stain Kit (Thermo Fisher Scientific Inc.) in the intracellular staining panels (6-7) following the manufacturer's instructions.

Supplementary Table 2. Target and reference gene primers

**Target Genes Primers**	
**Name**	**5'3'Sequence**
FOXP3-F	CAAGGGCCAAGGAAGGG
FOXP3-R	CCAGGCTGATCCTTTTCTGT
AATF-F	TGGAAAAGCAACTCTTCCTGA
AATF-R	ACAAAGGTGGCCCAGAATTT
GPI-F	GCTCGAAGTTGTCAAAACCC
GPI-R	AAACATGTTCGAGTTCTGGGA
PSMD9-F	TGCGGATCACTGTCACATTC
PSMD9-R	AGTTCGGCTCTGTGAACACC
TNFSF8-F	GGCCCATGACTTCTTGAATG
TNFSF8-R	AACTCACCTGACAACGTCCC
FCRH1-F	GCTATCAAAAACAGCTCGGC
FCRH1-R	AGGTACCATCCCTGACCTGG
UBASH3A-F	TGGAGTTCAGGTCTGTGGCT
UBASH3A-R	TTCCTGCCGGAAAACTACAC
BCL3-F	GCACCACAGCAATATGGAGA
BCL3-R	CCTATACCCCATGATGTGCC
SSTR3-F	GAGGGTGGTCAGCAGTCAG
SSTR3-R	GAGAGGGGACAGCAGAATGA
MAPK8IP1-F	AGCTGATGCCACACTCATCA
MAPK8IP1-R	CTTCGCCTCCCAATTTCAG
**Reference Genes Primers**	
**Name**	**5'3'Sequence**
B2M-F	TCTCTGCTGGATGACGTGAG
B2M-R	TAGCTGTGCTCGCGCTACT
TFRC-F	CCACCAAACAAGTTAGAGAATGC
TFRC-R	TGTGGGGAAGGGGCTGT
SDHA-F	TGGGAACAAGAGGGCATCTG
SDHA-R	CCACCACTGCATCAAATTCATG

Supplementary Table 2. Target and reference gene primers
Name and 5' and 3' sequences of the 10 target genes and 3 reference genes used.

Supplementary Table 3. Clinical and MRI outcomes

	Time periods			Statistical significance ǂ		
	Baseline	1 year	2 years	Baseline vs 1 year	1 year vs 2 years	Baseline vs 2 years
ARR »						
All	0.71+/-0.13	0.41+/-0.1	0.1+/-0.05	**p=0.0079**	**p<0.001**	**p<0.001**
NTZ group	0.07+/-0.06	0.45+/-0.16	0.09+/-0.07	**p=0.0026**	**p=0.0026**	p=0.5136
No NTZ group	1.02+/-0.22	0.42+/-0.14	0.07+/-0.06	**p=0.0011**	**p=0.0010**	**p<0.00109**
EDSS ¶						
All	2.63+/-1.59	2.68+/-1.73	2.41+/-1.59	p=0.8612	p=0.5353	p=0.412
NTZ group	3.17+/-1.68	3.39+/-1.72	3.3+/-1.54	p=0.8754	p=0.8545	p=0.9871
No NTZ group	2.18+/-1.4	2.09+/-1.53	1.71+/-1.27	p=0.5926	p=0.5396	p=0.2435
Number of GdE lesions »						
All	1.52+/-0.19	1.08+/-0.16	0.18+/-0.07	**p=0.0102**	**p<0.001**	**p<0.001**
NTZ group	0+/-0	0.69+/-0.2	0+/-0	**p<0.001**	**p<0.001**	**p**=0.9981
No NTZ group	1.59+/-0.27	1.27+/-0.24	0.31+/-0.12	**p**=0.0994	**p<0.001**	**p<0.001**
GdE lesion volume ¶						
All	0.27+/-0.89	0.22+/-0.56	0.03+/-0.16	p=0.6321	**p=0.0141**	**p=0.0429**
NTZ group	0+/-0	0.3+/-0.75	0+/-0	**p<0.001**	**p<0.001**	p=0.9900
No NTZ group	0.49+/-1.16	0.15+/-0.33	0.05+/-0.22	p=0.3823	p=0.2931	**p=0.0467**
Number of new T2-Weighted lesions »						
All	1.41+/-0.19	2+/-0.22	2.05+/-0.23	p=0.0881	p=0.4465	p=0.1532
NTZ group	0.78+/-0.21	1.91+/-0.33	0.09+/-0.07	**p<0.001**	**p<0.001**	**p<0.001**
No NTZ group	1.42+/-0.25	1.81+/-0.29	2.5+/-0.34	p=0.0683	**p=0.0142**	**p=0.0002**
T2-weighted lesion volume ¶						
All	9.52+/-7.17	9.64+/-6.85	9.84+/-7.51	p=0.8512	p=0.9498	p=0.9649
NTZ group	9.46+/-7.97	9.34+/-7.45	9.64+/-8.1	p=0.8831	p=0.9192	p=0.9869
No NTZ group	9.56+/-6.64	9.88+/-6.49	10+/-7.23	p=0.7126	p=0.8737	p=0.8815
T1-weighted lesion volume ¶						
All	1.06+/-1.36	1.07+/-1.32	1.1+/-1.38	p=0.4302	p=0.7467	p=0.7937
NTZ group	1.44+/-1.78	1.41+/-1.73	1.44+/-1.83	p=0.9731	p=0.8669	p=0.8842
No NTZ group	0.75+/-0.81	0.79+/-0.8	0.83+/-0.83	p=0.6429	p=0.9109	p=0.9839
Annual brain volume loss (BVL) ¶						
All	-	0.48+/-0.41	0.31+/-0.3	-	**p=0.0492**	-
NTZ group	-	0.45+/-0.4	0.3+/-0.31	-	**p=0.0394**	-
No NTZ group	-	0.5+/-0.43	0.33+/-0.3	-	**p=0.0406**	-
Percentage of relapse-free patients †£						
All	60	75	70	OR=2 (95%IC:0.76-5.2) p=0.2351	OR=5 (95%IC:1.27-19.6) **p=0.03**	OR=15 (95%IC:3.29-68.3) **p=0.0005**
NTZ group	94.44	66.6	61.12	OR= 0 (95% IC:0-0) p=0.06	OR=6 (95%IC:0.93-38.4) p=0.12	OR=1 p=1
No NTZ group	31.81	81.82	77.28	OR=12 (95%IC:2.43-59.2) **p=0.003**	OR=4 (95%IC:0.52-30.3) p=0.37	OR=~ **p=0.0001**
Percentage of CDP-free patients ‡£ ¥						
All	-	92.5	87.5	-	OR=1.5 (95%IC:0.25-8.8) p=1	-
NTZ group	-	95	92.5	-	OR=2 (95%IC:0.19-21.03) p=1	-
No NTZ group	-	97.5	95	-	OR=1 p=1	-
Percentage of patients with GdE lesions £						
All	17.5	22.5	2.94	OR= 1.5(95%IC:0.42-5.26) p=0.75	**OR= 0 (95%IC:0-0) p=0.0078**	**OR= 0 (95%IC:0-0) p=0.03**
NTZ group	0	22.2	0	OR=~ p=0.12	**OR= 0 (95%IC:0-0) p=0.02**	OR= ~ p=1
No NTZ group	31.81	22.72	5.26	OR= 0.5 (95%IC:0.09-2.63) p=0.68	**OR=0 (95%IC:0-0) p=0.02**	**OR= 0(95%IC:0-0) p=0.03**
Percentage of patients with T2w lesions £						
All	22.5	22.5	14.7	OR= 2(95%IC:0.19-21.03) p=1	OR=0.33 (95%IC:0.03-2.87) p=0.62	OR= 0(95%IC:0-~) p=1
NTZ group	11.1	16.6	6.66	OR=0.75 (95%IC:0.16-3.33) p=1	OR=0.5 (95%IC:0.94-2.63) p=0.68	OR=0.5 (95%IC:0.12-1.94) p=0.5
No NTZ group	31.81	27.27	21.05	OR=1 p=1	OR=0.42 (95%IC:0.11-1.59) p=0.34	OR=0.42 (95%IC:0.11-1.59) p=0.34
Percentage of patients with MRI activity £						
All	27.5	30	14.7	OR=1.6 (95%IC:0.4-6.86) p=0.72	**OR=0.2 (95%IC:0.05-0.78) p=0.03**	OR=0.33 (95%IC:0.09-1.15) p=0.14
NTZ group	11.11	27.7	6.66	OR= ~ p=0.25	OR= 0(95%IC:0-0) p=0.12	OR=0 (95%IC:0-~) p=1
No NTZ group	40.9	31.81	21.05	OR= 0.6 (95%IC:0.11-3.94) p=1	OR=0.33 (95%IC:0.07-1.52) p=0.28	OR=0.37 (95%IC:0.10-1.34) p=0.22

Supplementary Table 3. Clinical and MRI outcomes

Values of clinical and radiological variables that were measured at baseline, 1 and 2 years and statistical significance of the tests. ǂ For the numerical variables, p-values were calculated. For percentages, odds ratio, confidence interval and p-values were calculated. p<0.05 was considered statistically significant. » Count data for ARR, number of GdE lesions and number of new T2-weighted lesions were fitted to a zero-inflated Poisson model. Estimated ARR, number of GdE lesions and new T2-w lesions were calculated at different time periods (baseline, 1 year and 2 years). To assess the statistical significance between groups, we obtained the p-values by calculating the probability of observing a group rate given the other group rate as a null hypothesis. ¶ The values are the means+/-SD. P-values were calculated using the Wilcoxon signed-rank test to compare MRI and clinical measures before treatment and after 1 and 2 years. † Percentage estimates from the Kaplan-Meier cumulative survival curve for time to relapse. ‡ Percentage estimates from the Kaplan-Meier cumulative failure curve for time to confirmed disease progression. £ Odds ratio, confidence interval and p-values were calculated using the McNemar´s test to compare paired proportions. ¥ Patients were CPD-free at baseline as part of study design.

Supplementary Table 4. Comparison of lymphocyte subpopulations between patients and controls

	Percentages *			p-values ¶	
	Patients (Baseline) n=40	MS controls n=10	HCs n=10	Patients vs. MS controls	Patients vs. HCs
T Lymphocytes	73.98+/-9.71	73.23+/-7.91	75.5+/-4.64	*p=0.7342*	*p=0.8272*
Helper T cells	48.36+/-9.86	44.17+/-9.05	45.46+/-5.3	*p=0.1986*	*p=0.2072*
Cytotoxic T cells	22.02+/-5.84	25.32+/-6.93	25.41+/-4.94	*p=0.1524*	*p=0.0727*
B Lymphocytes	14.43+/-4.79	12.92+/-8.14	10.32+/-3.11	*p=0.1456*	*p=0.0079*
NK cells	9.44+/-7.94	11.05+/-3.16	10.07+/-2.98	*p=0.0896*	*p=0.1390*
NKT cells	3.2+/-3.73	3.08+/-42.17	3.3+/-1.22	*p=0.5605*	*p=0.0895*
Nkbright (% of NK)	12.58+/-8.04	11.24+/-6.35	8.79+/-4.15	*p=0.7342*	*p=0.1903*
Nkdim (% of NK)	86.86+/-7.88	88.75+/-6.35	91.20+/-4015	*p=0.6276*	*p=0.1326*
CD4 Naive T cells	23.01+/-9.33	19.18+/-6.37	21.99+/-5.65	*p=0.1986*	*p=0.7342*
CD4 Naive T cells	11.3+/-6.04	11.85+/-4.93	11.31+/-2.52	*p=0.6276*	*p=0.8462*
CD4 TCM cells	21.99+/-7.39	21.49+/-7.02	21.02+/-4.5	*p=1.0000*	*p=0.6276*
CD8 TCM cells	2.36+/-1.8	3.16+/-1.67	3.39+/-1.66	*p=0.0727*	*p=0.0495*
CD4 TEM cells	2.95+/-2.8	2.95+/-2.14	2.27+/-1.42	*p=0.716*	*p=0.6801*
CD4 TEMRA cells	0.41+/-0.78	0.54+/-0.71	0.17+/-0.15	*p=0.2347*	*p=0.4669*
CD8 TEM cells	2.71+/-2.15	2.65+/-1.17	3.18+/-2.18	*p=0.5605*	*p=0.3826*
CD8 TEMRA cells	5.66+/-4.23	7.67+/-1.9	7.52+/-2.92	*p=0.0257*	*p=0.0618*
Reg T cells	1.56+/-0.82	1.9+/-0.69	1.19+/-0.45	*p=0.2161*	*p=0.2072*
Naive B cells (% of CD20)	54.65+/-13	58+/-11.46	58.15+/-9.1	*p=0.7896*	*p=0.6624*
Mem B cells (% of CD20)	28.21+/-8.57	31.46+/-7.62	32.46+/-4.76	*p=0.3320*	*p=0.1456*
B1cells (% of CD20)	0.8+/-0.63	0.97+/-0.32	3.47+/-1.49	*p=0.1743*	***p<0.001***
B1cells CD11b (% of CD20)	0.72+/-0.52	0.87+/-0.28	3.21+/-1.55	*p=0.1149*	***p<0.001***
Naive B cells (% of CD19)	64.5+/-9.9	61.48+/-10.62	59.88+/-10.15	*p=0.3442*	*p=0.2859*
Mem B cells (% of CD19)	26.62+/-10.2	30.17+/-5.84	29.29+/-3.15	*p=0.1094*	*p=0.2859*
NoCS Mem B cells (% of CD19)	14.58+/-8.49	17.41+/-3.93	15.97+/-3.52	*p=0.1524*	*p=0.3200*
CS Mem B cells (% of CD19)	12.04+/-5.71	12.76+/-3.17	13.32+/-2.94	*p=0.3892*	*p=0.2859*
Immat B cells (% of CD19)	3.33+/-1.81	3.55+/-1.08	3.83+/-0.88	*p=0.3960*	*p=0.1327*
Transit B cells (% of CD19)	3.02+/-2.68	2.46+/-1.54	2.78+/-1.38	*p=0.8462*	*p=0.8084*
PB (% of CD19)	3.16+/-1.55	3.18+/-1.2	3.96+/-0.66	*p=0.8084*	*p=0.0653*
RegB1 (% of CD19)	14.09+/-10	18.41+/-7.64	18.58+/-9.34	*p=0.0851*	*p=0.0896*
RegB2 (% of CD19)	7.62+/-5.51	6+/-3.06	5.77+/-1.45	*p=0.6276*	*p=0.3631*
PC (% of CD19)	15.77+/-14.34	10+/-7.88	15.54+/-9.15	*p=0.0653*	*p=0.6105*
CD5+cells (% of CD19)	16.66+/-10.73	19.52+/-5.94	18.53+/-5.09	*p=0.1206*	*p=0.1456*
IL17+ cells	1.57+/-1.09	1.46+/-0.58	0.77+/-0.52	*p=0.8366*	*p=0.0186*
CD4+IL17+	0.99+/-0.6	0.95+/-0.39	0.58+/-0.42	*p=0.9013*	*p=0.0309*
CD8+IL17+	0.24+/-0.21	0.21+/-0.09	0.1+/-0.09	*p=0.5443*	*p=0.0226*
CD4+CCR6+CCR4+	2.7+/-2.45	2.93+/-1.37	2.15+/-1.93	*p=0.2538*	*p=0.4568*
CD4+CD161+CCR6+CCR4+	0.54+/-0.58	0.43+/-0.52	0.34+/-0.31	*p=0.6374*	*p=0.4419*
CD8+CCR6+CCR4+	10.72+/-4.43	13.85+/-5.08	12.42+/-6.98	*p=0.0825*	*p=0.5683*
CD8+CD161+CCR6+CCR4+	1.02+/-1.31	0.7+/-0.48	0.97+/-0.96	*p=0.6552*	*p=0.9210*
IFN+	20.53+/-10.72	21.27+/-4.6	20.05+/-6.61	*p=0.3210*	*p=0.7659*
IFN+CD4	7.18+/-3.72	6.89+/-1.92	6.46+/-2.7	*p=0.9802*	*p=0.5683*
IFN+CD8	9.74+/-6.56	11.15+/-4.31	10.91+/-5.98	*p=0.2436*	*p=0.4273*
IL2+	6.48+/-5.47	5.68+/-2.30	3.72+/-1.26	*p=0.8041*	*p=0.1928*
IL2+CD4	3.21+/-2.24	3.38+/-1.54	1.99+/-0.85	*p=0.5852*	*p=0.1574*
IL2+CD8	0.54+/-0.51	0.73+/-0.58	0.3+/-0.25	*p=0.3091*	*p=0.2975*
IL17+IFN+	0.3+/-0.33	0.28+/-0.1	0.16+/-0.13	*p=0.8620*	*p=0.1237*
IL17+IFN+CD4	0.12+/-0.09	0.13+/-0.06	0.09+/-0.09	*p=0.3853*	*p=0.2975*
IL17+IFN+CD8	0.17+/-0.15	0.1+/-0.06	0.05+/-0.04	*p=0.2861*	*p=0.0431*

Supplementary Table 4. Comparison of lymphocyte subpopulations between patients and controls

The percentages of 48 lymphocyte subpopulations were compared between the samples obtained from control subjects (naïve patients and healthy controls) and MS patients before fingolimod treatment. The percentages were calculated with respect to the total PBMCS or to other subpopulation, as specified in parentheses. The MS patients (naïve and no-naïve) exhibited a lower percentage of B1 cells than healthy controls. * The values are the mean +/- SD of each group. ¶ The p-values were calculated using the Mann-Whitney test to compare differences between subgroups. p<0.001 was considered statistically significant after Bonferroni´s correction for multiple tests.

Supplementary Table 5. Comparison of baseline lymphocyte subpopulations between the NTZ group and No NTZ group

	Subgroups *		P-Values ¶
	NTZ group (n=18)	No NTZ group (n=22)	NTZ vs. No NTZ group
T Lymphocytes	73.4+/-10.97	74.46+/-8.79	*p=0.8704*
Helper T cells	47.55+/-11.41	49.03+/-8.61	*p=0.8278*
Cytotoxic T cells	22.08+/-5.92	21.98+/-5.91	*p=0.7649*
B Lymphocytes	14.17+/-5.19	14.65+/-4.54	*p=0.5681*
NK cells	10.38+/-6.73	8.66+/-8.89	*p=0.0645*
NKT cells	4.21+/-4.19	2.37+/-3.17	*p=0.0625*
Nkbright (% of NK)	12.86+/-8.27	12.91+/-8.05	*p=0.9783*
Nkdim (% of NK)	87.14+/-8.27	86.65+/-7.74	*p=0.8490*
CD4 Naive T cells	21.85+/-11.6	23.96+/-7.12	*p=0.2212*
CD4 Naive T cells	8.66+/-5.5	13.46+/-5.69	*p=0.0060*
CD4 TCM cells	22.6+/-7.46	21.49+/-7.48	*p=0.4465*
CD8 TCM cells	2.35+/-1.66	2.36+/-1.94	*p=0.7649*
CD4 TEM cells	2.9+/-1.99	3+/-3.37	*p=0.3277*
CD4 TEMRA cells	0.2+/-0.34	0.58+/-0.99	*p=0.4465*
CD8 TEM cells	3.65+/-2.62	1.94+/-1.27	*p=0.0388*
CD8 TEMRA cells	7.42+/-4.75	4.22+/-3.18	*p=0.0134*
Reg T cells	1.4+/-0.83	1.69+/-0.8	*p=0.2212*
Naive B cells (% of CD20)	51.34+/-12.9	57.36+/-12.73	*p=0.1212*
Mem B cells (% of CD20)	29.13+/-7.23	27.46+/-9.63	*p=0.5680*
B1cells (% of CD20)	0.73+/-0.37	0.86+/-0.79	*p=0.6341*
B1cells CD11b (% of CD20)	0.67+/-0.32	0.75+/-0.65	*p=0.5498*
Naive B cells (% of CD19)	60.69+/-9.32	67.61+/-9.45	*p=0.0240*
Mem B cells (% of CD19)	29.65+/-10.5	24.15+/-9.47	*p=0.0428*
NoCS Mem B cells (% of CD19)	17.72+/-9.07	12.01+/-7.22	*p=0.0363*
CS Mem B cells (% of CD19)	11.93+/-6	12.14+/-5.6	*p=0.9350*
Immat B cells (% of CD19)	2.88+/-1.43	3.69+/-2.03	*p=0.2650*
Transit B cells (% of CD19)	4.59+/-3.15	1.7+/-1.31	***<p=0.001***
PB (% of CD19)	3.32+/-1.57	3.02+/-1.56	*p=0.3769*
RegB1 (% of CD19)	16.7+/-12.03	11.96+/-7.61	*p=0.1575*
RegB2 (% of CD19)	7.68+/-6.55	7.57+/-4.65	*p=0.8491*
PC (% of CD19)	15.59+/-18.52	15.91+/-10.2	*p=0.3277*
CD5+cells (% of CD19)	16.97+/-15.23	16.4+/-5.1	*p=0.4629*
IL17+ cells	1.26+/-0.92	1.82+/-1.17	*p=0.1574*
CD4+IL17+	0.84+/-0.54	1.11+/-0.62	*p=0.1740*
CD8+IL17+	0.17+/-0.17	0.29+/-0.23	*p=0.0868*
CD4+CCR6+CCR4+	3.64+/-3.34	1.96+/-1.03	*p=0.1486*
CD4+CD161+CCR6+CCR4+	0.62+/-0.68	0.47+/-0.51	*p=0.5520*
CD8+CCR6+CCR4+	11.41+/-5.13	10.19+/-3.86	*p=0.4445*
CD8+CD161+CCR6+CCR4+	1.4+/-1.85	0.73+/-0.55	*p=0.1408*
IFN+	22.86+/-11.79	18.74+/-9.72	*p=0.2882*
IFN+CD4	7.35+/-3.68	7.06+/-3.82	*p=0.6302*
IFN+CD8	12.42+/-8.34	7.67+/-3.82	*p=0.0474*
IL2+	7.31+/-6.5	5.83+/-4.38	*p=0.6420*
IL2+CD4	3.4+/-2.03	3.03+/-2.47	*p=0.4469*
IL2+CD8	0.47+/-0.33	0.6+/-0.63	*p=0.9775*
IL17+IFN+	0.24+/-0.16	0.35+/-0.42	*p=0.4273*
IL17+IFN+CD4	0.09+/-0.09	0.14+/-0.09	*p=0.0443*
IL17+IFN+CD8	0.14+/-0.14	0.19+/-0.16	*p=0.1567*

Supplementary Table 5. Comparison of baseline lymphocyte subpopulations between the NTZ group and No NTZ group

The percentages of 48 lymphocyte subpopulations were compared between samples from NTZ and No NTZ patients. The percentages were calculated with respect to the total PBMCS or to other subpopulation, as specified in parentheses. The only significantly different subpopulation was the transitory B cells, for which a lower percentage was observed in the NTZ-group
* The values are the mean +/- SD of each group. ¶ The p-values were calculated using the Mann-Whitney test to compare differences between subgroups. $p<0.001$ was considered statistically significant after Bonferroni´s correction for multiple tests.

Supplementary Table 6. Effects of fingolimod on lymphocyte subpopulations after 6 months of treatment

Lymphocyte subpopulations	Porcentajes			p-values (pre vs post-tratamiento)		
	**Pre-tratamiento**	**Post-tratamiento**	**Fold-change**	**Todos los pacientes N=40**	**Grupo NTZ N=18**	**Grupo no-NTZ N=22**
Linfocitos T (LT)	73,98+/-8,7	43,15+/-18,25	-1,71	<0.001	<0.001	<0.001
Células T colaboradoras (Th)	48,36+/-9,17	14,51+/-10,06	-3,33	<0.001	<0.001	<0.001
Células T citotóxicas (Tc)	22,02+/-6,01	22,13+/-11,31	1,00	0.9940	0.2343	0.2046
Linfocitos B (LB)	14,43+/-5,39	6,85+/-3,5	-2,10	<0.001	0.0013	<0.001
Células asesinas naturales (NK)	9,44+/-6,7	43,17+/-18,05	4,57	<0.001	<0.001	<0.001
Células T NK (NKT)	3,2+/-3,19	11,37+/-11,32	3,55	<0.001	<0.001	<0.001
NKbright (% de NKs)	12,89+/-7,34	5,03+/-2,89	-2,56	<0.001	0.0013	<0.001
NKdim (% de NKs)	86,87+/-7,25	94,67+/-2,85	1,08	<0.001	0.0016	0.0024
CD4 T naïve (CD4 Tn)	23,01+/-8,41	2,55+/-2,73	-9,02	<0.001	<0.001	<0.001
CD8 T naïve (CD8 Tn)	11,3+/-5,37	1,85+/-1,72	-6,10	<0.001	<0.001	<0.001
CD4 T de memoria central (CD4 TCM)	21,99+/-6,85	6,08+/-4,62	-3,61	<0.001	<0.001	<0.001
CD8 T de memoria central (CD8 TCM)	2,36+/-1,78	1,39+/-1,55	-1,69	<0.001	0.0017	<0.001
CD4 T de memoria efectora (CD4 TEM)	2,95+/-2,5	4,96+/-5,03	1,68	0.0074	0.0066	0.0017
CD4 T de memoria efectora RA (CD4 TEM)	0,41+/-0,71	0,91+/-1,37	2,21	<0.001	<0.001	0.0064
CD8 T de memoria efectora (CD8 TEM)	2,71+/-2	6,75+/-7,42	2,49	<0.001	0.0072	0.0030
CD8 T de memoria efectora RA (CD8 TEM)	5,66+/-3,81	12,14+/-7,08	2,14	<0.001	0.0023	<0.001
T reguladoras (T reg)	3,2+/-1,36	7,03+/-4,2	2,19	<0.001	0.0016	<0.001
B naïve (Bn1) (% de CD20)	54,65+/-12,13	72,78+/-13,51	1,33	<0.001	<0.001	<0.001
B de memoria (BMem1) (% de CD20)	28,21+/-8	15,71+/-7,19	-1,79	<0.001	<0.001	<0.001
Células LB1cells (% deCD20)	0,8+/-1,27	2,69+/-2,15	3,36	<0.001	<0.001	<0.001
Células LB1 CD11b+ (% de CD20)	0,72+/-1,19	0,84+/-0,74	1,16	0.6133	0.4380	0.3570
B naïve (Bn) (% de CD19)	64,5+/-10,06	81,2+/-7,61	1,25	<0.001	<0.001	<0.001
B de memoria (BMem2) (% de CD19)	26,62+/-8,81	11,53+/-6,69	-2,30	<0.001	<0.001	<0.001
B de memoria class switched (CS BMem) (% de CD19)	14,58+/-7,29	6,17+/-5,2	-2,36	<0.001	0.0019	0.0033
B de memoria non-class switched (NoCS BMem) (% de CD19)	12,04+/-4,96	5,47+/-2,75	-2,20	<0.001	0.0013	<0.001
Células B inmaduras (B inm) (% de CD19)	3,33+/-1,58	5,73+/-2,95	1,72	<0.001	0.0096	0.0033
Células B transicionales (BTransi) (% de CD19)	3,02+/-2,68	10,38+/-6,36	3,20	<0.001	0.0030	<0.001
Plasmablastos (PB) (% de CD19)	2,93+/-1,45	6,7+/-3,92	2,28	<0.001	0.0023	0.0019
Células B reguladoras (Breg1) (% de CD19)	2,47+/-2,02	1,15+/-1,11	-2,14	<0.001	0.0013	0.0037
Células B reguladoras (Breg1) (% de CD19)	1,33+/-0,94	0,61+/-0,63	-2,18	0.0012	0.0028	0.0037
Células plasmáticas (CP) (% de CD19)	15,77+/-12,76	14,7+/-13,46	-1,07	0.6271	0.6529	0.8484
Células B CD5+ (% de CD19)	16,66+/-9,32	27,8+/-9,11	1,66	<0.001	0.0017	<0.001
Células IL17+	1,57+/-0,98	0,54+/-0,41	-2,90	<0.001	0.0032	<0.001
Células CD4+IL17+	0,99+/-0,55	0,28+/-0,23	-3,53	<0.001	<0.001	<0.001
Células CD8+IL17+	0,24+/-0,19	0,1+/-0,11	-2,4	<0.001	0.0017	<0.001
Células CD4+CCR6+CCR4+	2,7+/-2,2	1,61+/-1,87	-1,67	0.0045	0.0067	0.0065
Células CD4+CD161+CCR6+CCR4+	0,54+/-0,54	0,43+/-0,44	-1,25	0.8137	0.2078	0.1808
Células CD8+CCR6+CCR4+	10,72+/-5,09	10,77+/-6,24	1,00	0.8752	0.5245	0.9032
Células CD8+CD161+CCR6+CCR4+	1,02+/-1,15	2,08+/-1,38	2,03	<0.001	0.0055	<0.001
Células IFN+	20,53+/-9,25	10,3+/-3,63	-1,99	<0.001	<0.001	0.0015
Células IFN+CD4	7,18+/-3,29	1,56+/-1,33	-4,60	<0.001	<0.001	<0.001
Células IFN+CD8	9,74+/-6,09	4,27+/-2,27	-2,28	<0.001	<0.001	<0.001
Células IL2+	6,48+/-4,66	2,62+/-2,82	-2,47	<0.001	0.0023	0.0014
Células IL2+CD4	3,21+/-2	0,86+/-0,91	-3,73	<0.001	<0.001	<0.001
Células IL2+CD8	0,54+/-0,5	0,54+/-0,83	1	0.2387	0.6417	0.1790
Células IL17+IFN+	0,3+/-0,28	0,41+/-0,3	1,36	0.0077	0.0554	0.0536
Células IL17+IFN+CD4	0,12+/-0,09	0,11+/-0,1	-1,09	0.5717	0.5245	0.9032
Células IL17+IFN+CD8	0,17+/-0,13	0,15+/-0,12	-1,13	0.8382	0.1354	0.6143

Supplementary Table 6. Effects of fingolimod on lymphocyte subpopulations after 6 months of treatment
The percentages of 48 lymphocyte subpopulations were compared between the samples obtained from MS patients before and after 6 months of fingolimod treatment. The percentages were calculated with respect to the total PBMCs or to another subpopulation, as specified in parentheses. Most lymphocyte subpopulations were modified by fingolimod in this study*. The values are the means +/- SD at each time interval. ¶ All values were calculated as the ratio between the final value and the initial value. A positive or negative sign indicates an increase or decrease, respectively. ǂ The p-values were calculated using the Wilcoxon signed-rank test to compare differences in flow cytometry data before and after 6 months of therapy. p<0.001 was considered statistically significant after Bonferroni´s correction for multiple tests.

Supplementary table 7. Differentially expressed genes (DEGs) before treatment in responder vs non-responder patients.

Clinical response at 1 year

	Next generation RNA sequencing (RNA-seq)					q-PCR			
	Mean of expression levels		log2 FC ǂ	p-value*	p-value adj ~	Relative expression ratio †		FC ¶	p-value ‡
	NEDA patients	EDA patients				NEDA patients	EDA patients		
FOXP3	3.26+/-0.42	1.67+/-0.49	1.14	1.48E-05	0.007	0.0099+/-0.0082	0.0013+/-0.0015	7.46	0.001
AATF	25.8+/-2.06	18.71+/-0.45	0.46	1.68E-05	0.007	0.0220+/-0.0094	0.0195+/-0.0314	1.12	0.047
CXCL9	7.93+/-2.32	0.44+/-0.89	4.15	1.47E-05	0.007				
GPI	25.6+/-2.78	18.4+/-0.84	0.47	0.00016	0.03	2.5499+/-3.5412	1.940+/-2.6596	1.31	0.023
PSMD9	18.29+/-1.08	12.88+/-1.44	0.68	0.00044	0.048	6.3500+/-0.1652	4.8713+/-5.8970	1.3	0.063
TNFSF8	84.69+/-18.07	43.51+/-17.92	1.01	0.00033	0.044	2.0279+/-2.1090	1.1060+/-1.1371	1.83	0.001
IL21	55.97+/-40.57	16.28+/-6.88	1.78	0.00041	0.048				
IFNG	8628.55+/-3645.31	3857.11+/-689.08	1.21	0.00026	0.038				
IL17A	32.06+/-10.47	8.35+/-3.88	0.46	2.43E-05	0.008				
FCRL1	0.74+/-0.31	2.79+/-1.33	-1.88	7.78E-06	0.005	0.0610+/-0.0656	0.1521+/-0.1718	-2.49	0.048
UBASH3A	1.01+/-0.18	2.72+/-1.33	-1.45	1.20E-05	0.006	0.2476+/-0.2377	0.1900+/-0.2422	1.3	0.051
BCL3	3.13+/-0.83	7.5+/-2.58	-1.33	2.20E-05	0.008	0.0144+/-0.0239	0.0406+/-0.0374	-2.82	0.032
SSTR3	0.39+/-0.11	0.97+/-0.37	-1.16	1.37E-05	0.019	0.0002+/-0.0001	0.0008+/-0.0010	-3.60	0.032
MAPK8IP1	0.55+/-0.09	1.03+/-0.26	-1.17	4.93E-05	0.014	0.0010+/-0.0037	0.0020+/-0.0018	-1.93	0.002
FCRL2	0.35+/-0.32	1.55+/-0.76	-2.16	9.19E-05	0.023				
LEP	0.16+/-0.07	0.74+/-0.69	-3.1	0.00018	0.034				
NLRP1	9.83+/-2.19	17.38+/-3.99	-0.82	0.00036	0.046				

Clinical response at 2 years

	Next generation RNA sequencing (RNA-seq)				
	Mean of expression levels		log2 FC ǂ	p-value*	p-value adj ~
	NEDA patients	EDA patients			
FUT1	1.11+/-0.24	0.59+/-0.14	2.42	3.83E-07	0.001
IFNG	9322.2+/-3809.21	4189.92+/-1021.97	1.31	1.61E-05	0.015
SPP1	70.1+/-21.08	22.2+/-13.83	1.74	2.75E-05	0.021
SLC4A4	0.42+/-0.1	0.15+/-0.06	1.62	3.60E-05	0.025
B3GAT1	2.13+/-0.95	0.74+/-0.37	1.48	5.94E-05	0.033
RASIP1	0.5+/-0.13	0.18+/-0.09	1.60	5.92E-05	0.033
TNFRSF9	60.15+/-21.29	23.87+/-9.71	1.43	6.10E-05	0.033
MUM1	1.09+/-0.18	2.02+/-0.58	-0.96	0.00010	0.045
EGR4	6.56+/-1.08	16.65+/-6.6	-1.51	4.92E-08	0.000
NFKBIZ	83.85+/-7.28	137.42+/-16.45	-0.69	1.07E-06	0.003
CYP4V2	3.57+/-0.16	6.25+/-1.5	-1.77	1.38E-06	0.003
TCL1A	0.47+/-0.31	2.65+/-1.75	-2.61	2.62E-06	0.005
AFF3	2.19+/-0.59	5.19+/-1.7	-1.35	8.33E-06	0.011
DHRS1	0.94+/-0.12	1.82+/-0.4	-1.14	1.30E-05	0.014
CCDC88B	6.99+/-2.03	11.28+/-1.53	-0.83	3.19E-05	0.024
MMP2	0.06+/-0.04	0.41+/-0.29	-2.67	7.13E-05	0.037
ADORA2A	14.2+/-3.75	19.7+/-4.92	-0.82	8.96E-05	0.042
JAK3	7.43+/-1.14	13.11+/-2.81	-1.00	8.86E-05	0.042

Supplementary table 7. Differentially expressed genes (DEGs) before treatment in responder vs non-responder patients.

In samples collected before treatment, the gene expression levels of responder vs non-responder patients (at 1 and 2 years) were compared. In this table, the most representative DEGs in responder patients at 1 and 2 years are shown, as well as the q-PCR validation. ǂ The log2 (fold-change) or log2FC is the log-ratio of a gene expression values in two different biological conditions, in this case NEDA status vs EDA status. * The estimated significance level (p-value) of differential gene expression was calculated with DESeq2, which uses a negative binomial distribution. ~ p-values were corrected to account for multiple hypotheses testing using Benjamini and Hochberg false discovery rate (FDR) adjustment. Genes with an FDR less than or equal to 0.05 were selected as differentially expressed. † Relative expression of the target genes versus three reference genes using the $2^{-\Delta\Delta Ct}$ method. ¶ Fold change is the ratio of the gene expression values. ‡ p-value was calculated using the t-test to compare means of expression levels.

Supplementary Table 8. Significantly enriched gene sets (GSEA)

	ES	NES	nominal p-value	FDR
BIOCARTA_BCELLSURVIVAL_PATHWAY	0.27	1.30	0.0020	0.0237
BIOCARTA_CCR3_PATHWAY	0.29	1.48	0.0655	0.2037
BIOCARTA_CCR5_PATHWAY	0.25	1.24	0.0412	0.1861
BIOCARTA_CXCR4_PATHWAY	0.25	1.41	0.0000	0.0037
BIOCARTA_CYTOKINE_PATHWAY	-0.30	-1.65	0.0082	0.0874
BIOCARTA_ERK_PATHWAY	0.27	1.60	0.0493	0.1391
BIOCARTA_GATA3_PATHWAY	-0.27	-1.29	0.0663	0.1369
BIOCARTA_IL10_PATHWAY	0.32	1.60	0.0332	0.1401
BIOCARTA_IL12_PATHWAY	0.11	0.62	0.0663	0.1369
BIOCARTA_IL17_PATHWAY	-0.21	-1.00	0.0409	0.1564
BIOCARTA_INFLAM_PATHWAY	-0.18	-1.18	0.2087	0.1619
BIOCARTA_MAPK_PATHWAY	0.23	2.52	0.0000	0.0021
BIOCARTA_NKCELLS_PATHWAY	0.61	3.26	0.0000	0.0000
BIOCARTA_NKT_PATHWAY	-0.17	-1.06	0.0480	0.1252
BIOCARTA_PYK2_PATHWAY	0.20	1.24	0.0020	0.0237
BIOCARTA_RAC1_PATHWAY	0.28	1.58	0.0491	0.1437
BIOCARTA_RAS_PATHWAY	0.35	2.01	0.0000	0.0295
BIOCARTA_RELA_PATHWAY	0.28	1.36	0.0123	0.2372
BIOCARTA_RHO_PATHWAY	0.41	2.70	0.0000	0.0013
BIOCARTA_TGFB_PATHWAY	0.34	1.81	0.0155	0.0717
BIOCARTA_TH1TH2_PATHWAY	-0.18	-0.92	0.0560	0.2463
KEGG_ANTIGEN_PROCESSING_AND_PRESENTATION	0.20	2.07	0.0059	0.0108
KEGG_APOPTOSIS	0.15	1.66	0.0207	0.0654
KEGG_B_CELL_RECEPTOR_SIGNALING_PATHWAY	0.20	2.02	0.0084	0.0136
KEGG_COMPLEMENT_AND_COAGULATION_CASCADES	0.25	2.25	0.0000	0.0045
KEGG_CYTOKINE_CYTOKINE_RECEPTOR_INTERACTION	0.12	2.28	0.0000	0.0042
KEGG_FC_GAMMA_R_MEDIATED_PHAGOCYTOSIS	0.30	3.35	0.0000	0.0000
KEGG_MAPK_SIGNALING_PATHWAY	0.12	2.08	0.0042	0.0109
KEGG_MELANOMA	0.21	1.97	0.0085	0.0179
KEGG_NATURAL_KILLER_CELL_MEDIATED_CYTOTOXICITY	0.24	3.15	0.0000	0.0000
KEGG_OXIDATIVE_PHOSPHORYLATION	-0.13	-1.68	0.0402	0.0920
KEGG_SPHINGOLIPID_METABOLISM	0.25	1.80	0.0204	0.0404
KEGG_T_CELL_RECEPTOR_SIGNALING_PATHWAY	0.11	1.35	0.1269	0.1913
KEGG_TGF_BETA_SIGNALING_PATHWAY	0.14	1.52	0.0671	0.1045
REACTOME_ACTIVATION_OF_NF_KAPPAB_IN_B_CELLS	-0.18	-1.66	0.0333	0.0574
REACTOME_ANTIGEN_PROCESSING_CROSS_PRESENTATION	0.15	1.54	0.0565	0.1139
REACTOME_APOPTOSIS	0.07	0.92	0.0513	0.2377
REACTOME_CD28_CO_STIMULATION	0.19	1.31	0.1630	0.2426
REACTOME_CLASS_I_MHC_MEDIATED_ANTIGEN_PROCESSING_PRESENTATION	0.10	1.74	0.0195	0.0556
REACTOME_COMPLEMENT_CASCADE	0.32	1.83	0.0139	0.0394
REACTOME_DOWNSTREAM_SIGNALING_EVENTS_OF_B_CELL_RECEPTOR_BCR	-0.08	-0.91	0.0502	0.2355
REACTOME_ERK_MAPK_TARGETS	0.31	1.72	0.0208	0.0612
REACTOME_IL_2_SIGNALING	0.23	1.73	0.0295	0.0590
REACTOME_INNATE_IMMUNE_SYSTEM	0.20	3.46	0.0000	0.0000
REACTOME_MHC_CLASS_II_ANTIGEN_PRESENTATION	0.20	2.22	0.0041	0.0060
REACTOME_NCAM1_INTERACTIONS	-0.15	-1.02	0.0082	0.0874
REACTOME_PI3K_AKT_ACTIVATION	0.20	1.43	0.0988	0.1686
REACTOME_SIGNALING_BY_RHO_GTPASES	0.28	3.37	0.0000	0.0000
REACTOME_SIGNALING_BY_TGF_BETA_RECEPTOR_COMPLEX	0.09	0.83	0.0663	0.1369
REACTOME_SIGNALING_BY_THE_B_CELL_RECEPTOR_BCR	0.05	0.63	0.0041	0.0060
REACTOME_SIGNALLING_TO_ERKS	0.32	2.25	0.0000	0.0053
REACTOME_SIGNALLING_TO_RAS	0.32	1.97	0.0062	0.0231
REACTOME_SPHINGOLIPID_METABOLISM	0.28	2.52	0.0000	0.0010

Supplementary Table 7. Significantly enriched gene sets (GSEA)
Enrichment score (ES), normalized enrichment score (NES), nominal p-value and false discovery rate (FDR) of pathways related to multiple sclerosis, autoimmunity and fingolimod mechanisms of action that achieved significant enrichment in GSEA (FDR q-values < 0.25).

Supplementary table 9. Posterior predictive response in model validation

Sample	Posterior predictive mean	Posterior predictive lower 95%	Posterior predictive upper 95%	Real response
1166	0,014973171	1,24E-04	0,08193146	0
1175	0,864864671	5,75E-01	0,99025255	1
1198	0,011318419	1,31E-08	0,09943895	0
1172	0,145597607	9,71E-03	0,46624478	0
1174	0,004772552	4,51E-06	0,03063313	0
1254	0,238005954	7,14E-03	0,65438967	0
1301	0,033617637	6,29E-07	0,27805955	0
1315	0,844556423	4,85E-01	0,99252926	0
1330	0,893776563	5,57E-01	0,99724563	1
1354	0,015081467	1,76E-09	0,16324828	0
1564	0,03029961	1,18E-07	0,27908216	0
1571	0,183560574	1,74E-02	0,55312179	0
1595	0,073613074	4,90E-04	0,31758959	0
1598	0,008079493	8,71E-08	0,07094498	0
1627	0,003240338	1,86E-11	0,0278112	0
1630	0,578044005	2,27E-01	0,86406943	0
1631	0,018487652	8,83E-05	0,09886409	0
1655	0,559918281	7,94E-02	0,97243389	1
1666	0,203399182	4,04E-02	0,45546709	0
1699	0,603018825	2,52E-01	0,89530839	0
1706	0,921410004	7,13E-01	0,99623156	1
1725	0,108755784	9,80E-04	0,51386938	0
1729	0,972366882	7,99E-01	0,99996937	1
1731	0,960468038	7,52E-01	0,99985463	1
1739	0,844811946	3,23E-01	0,99823225	1
1744	0,001923548	8,49E-13	0,01758241	0
1745	0,006102073	3,57E-06	0,04538053	0
1874	0,05124762	1,24E-03	0,22009736	0
1934	0,041527435	1,10E-03	0,17619654	0

Supplementary table 9. Posterior predictive response in model validation.
The table shows the mean, lower and upper posterior predictive for each sample in the validation. Real response is 0 for non-responder and 1 for responder patients (NEDA-4 at 2 years).

Supplementary Figure S1. Gating strategy.

(a, b in S1.1-S1.7) Sample data from a control are shown. The lymphocyte population was gated on a forward scatter (FSC)/side scatter (SSC) plot. Live lymphocytes were further gated by 4′,6-diamidine-2′-phenylindole dihydrochloride (DAPI) in S1.1-S1.5 and by the fluorescent reactive dye from the LIVE/DEAD™ Fixable Dead Cell Stain Kit (Termo Fisher Scientific Inc.) in S1.6-S1.7.

S1.1

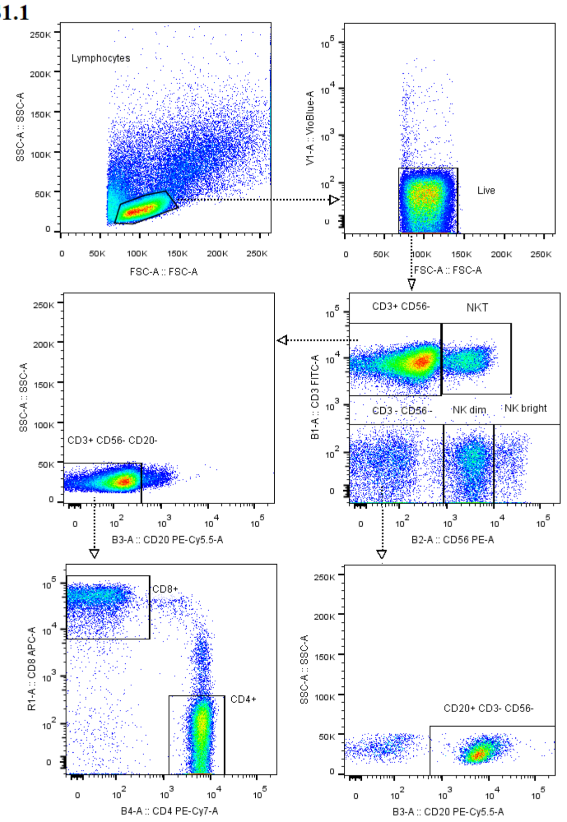

S1.1 Gating strategy in panel 1: (c) Live lymphocytes were then further gated to determine CD3$^-$CD56$^-$, CD3$^-$CD56dim (NKdim), CD3$^-$CD56bright (NKbright), CD3$^+$CD56$^-$ and CD3$^+$CD56$^+$ (NKT). **(d)** CD20$^-$ from CD3$^+$CD56$^-$ (T lymphocytes). **(e)** CD4$^+$ (Helper T cells) and CD8$^+$ (cytotoxic T cells) from T lymphocytes and (**f**) CD20$^+$ cells from CD3$^-$CD56$^-$ (B lymphocytes).

S1.2

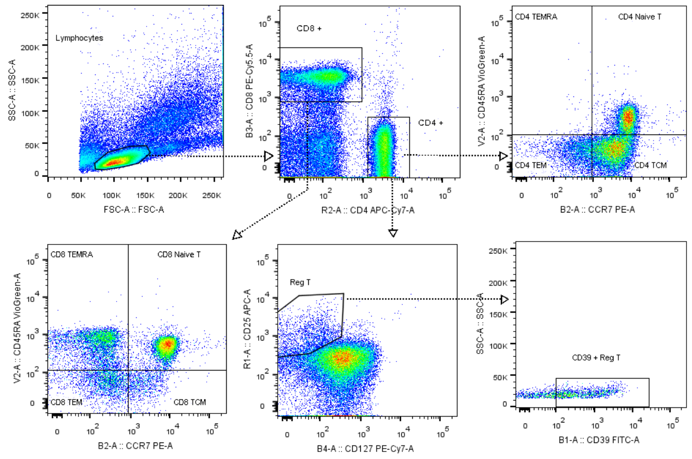

S1.2 Gating strategy in panel 2: (c) Live lymphocytes were then further gated to determine CD4$^+$ (Helper T cells) and CD8$^+$ (cytotoxic T cells), **(d)** CCR7$^-$CD45RA$^-$ (CD4TEM cells), CCR7$^+$CD45RA$^-$ (CD4TCM cells), CCR7$^-$CD45RA$^+$ (CD4TEMRA cells), and CCR7$^+$CD45RA$^+$ (CD4 naïve T cells) from CD4+ cells. **(e)** CCR7$^-$CD45RA$^-$ (CD8TEM cells), CCR7$^+$CD45RA$^-$ (CD8TCM cells), CCR7$^-$CD45RA$^+$ (CD8TEMRA cells), and CCR7$^+$CD45RA$^+$ (CD8 naïve T cells) from CD8+ cells. (**f**) CD127^{down}CD25^{high} from CD4 cells (regulatory T cells). **(g)** CD39$^+$ cells from regulatory T cells.

S1.3

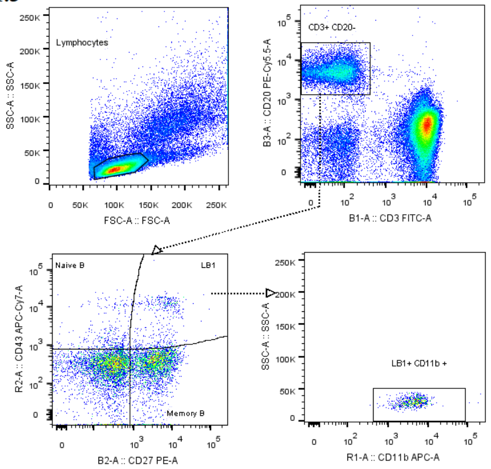

S1.3 Gating strategy in panel 3: (c) Live lymphocytes were then further gated to evaluate CD3⁻CD20⁺ cells, **(d)** CD27⁺CD43⁻ (memory1 B cells), CD27⁻CD43⁺ (naïve 1 B cells), and CD27⁺CD43⁺ (LB1 cells) from CD3⁻CD20⁺ cells **(e)** CD11b+ cells from LB1 cells.

S1.4

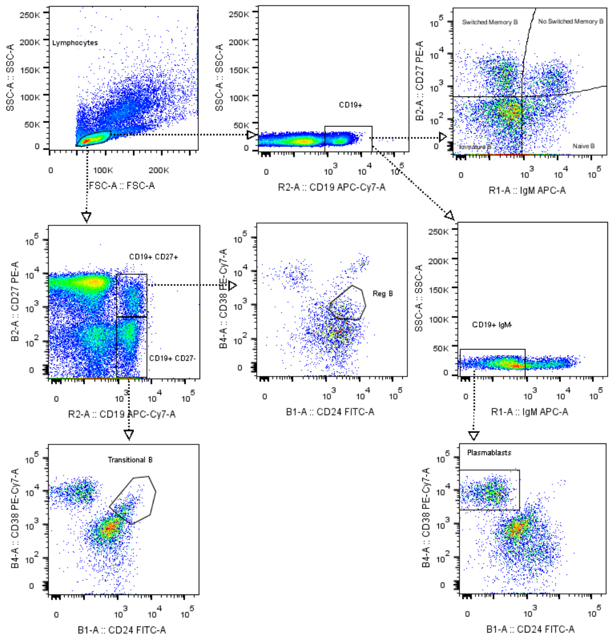

S1.4 Gating strategy in panel 4: (c) Live lymphocytes were then further gated to determine CD19+ cells, **(d)** IgM⁻CD27⁻ (transitory B cells), IgM ⁺ CD27⁻ (naïve 2 B cells), IgM ⁻ CD27⁺, (switched memory B cells), and IgM ⁺ CD27⁺ (non-switched memory B cells) from CD19+ cells. **(e)** IgM ⁻ cells from CD19⁺. **(f)** CD24⁻CD38^{high} cells from CD19⁺IgM⁻ (plasmablasts). **(g)** CD24⁻CD38^{high} cells from CD19⁺IgM⁻ (plasmablasts). **(h)** CD19⁺CD27⁻ and CD19⁺CD27⁺ from live lymphocytes. **(i)** CD24^{high} CD38^{int} from CD19⁺CD27⁺ (regulatory B cells) and **(j)** CD24^{high}CD38^{high} from CD19⁺CD27⁻ (transitory B cells).

S1.5

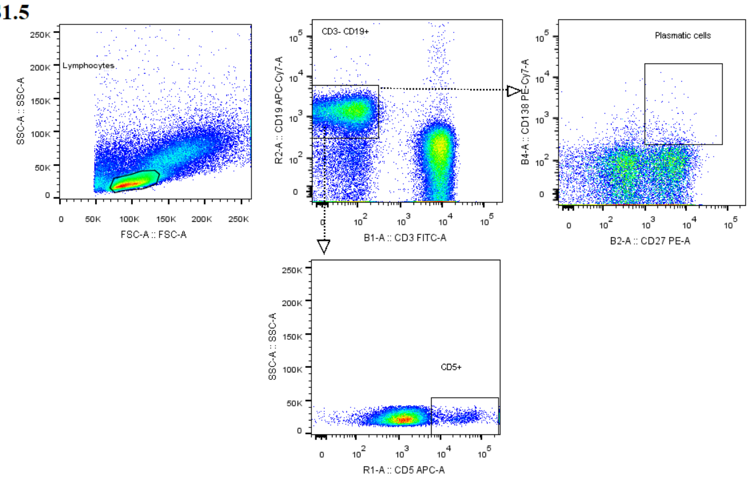

S1.5 Gating strategy in panel 5: (c) Live lymphocytes were then further gated to evaluate CD19+CD3⁻cells and **(d)** CD138⁺CD27⁺ cells from CD19+CD3⁻cells (plasmatic cells). **(e)** CD5⁺ cells from CD19+CD3⁻cells.

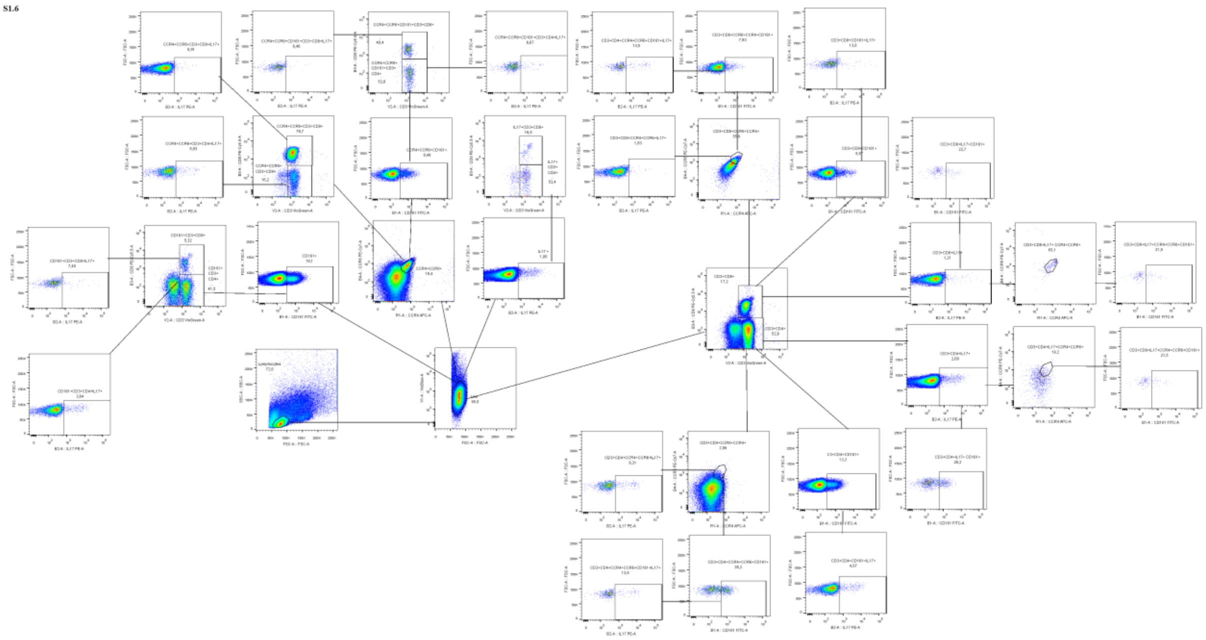

S1.6 Gating strategy in panel 6. All possible combinations between surface (CD3, CD8, CD161, CCR6 and CCR4) and intracellular (IL-17) markers for Th17 cells allowing us define the percentage of IL-17-producing cells among Th17 cells measured by surface markers.

S1.7

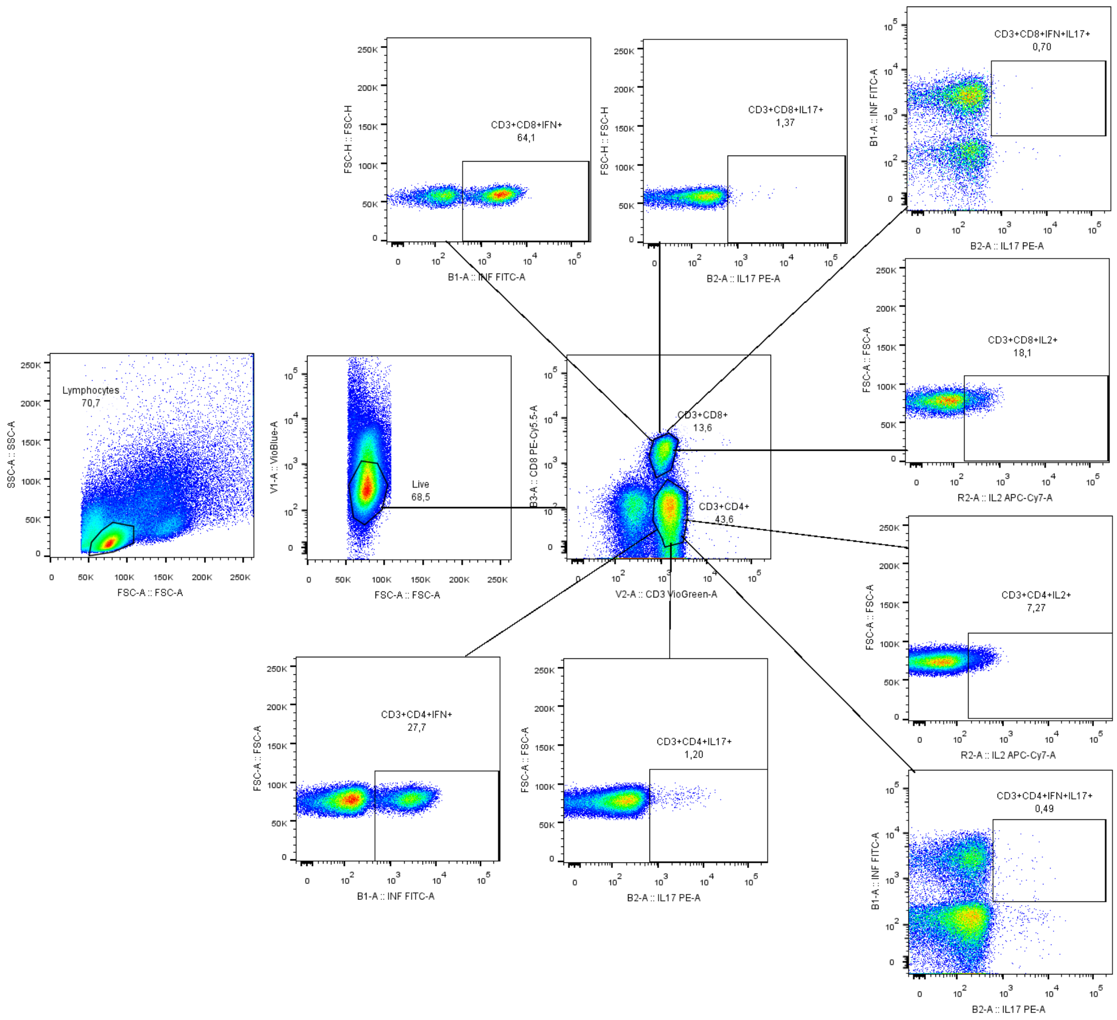

S1.7 Gating strategy in panel 7: All possible combinations between surface (CD3 and CD8) and intracellular (IL-17, IL-2 and IFN) markers allowing us to define the percentages of IL-17, IL-2 and IFN-producing cells among helper T and cytotoxic T cells.

Supplementary Figure S2. Lymphocyte subpopulation correlations.

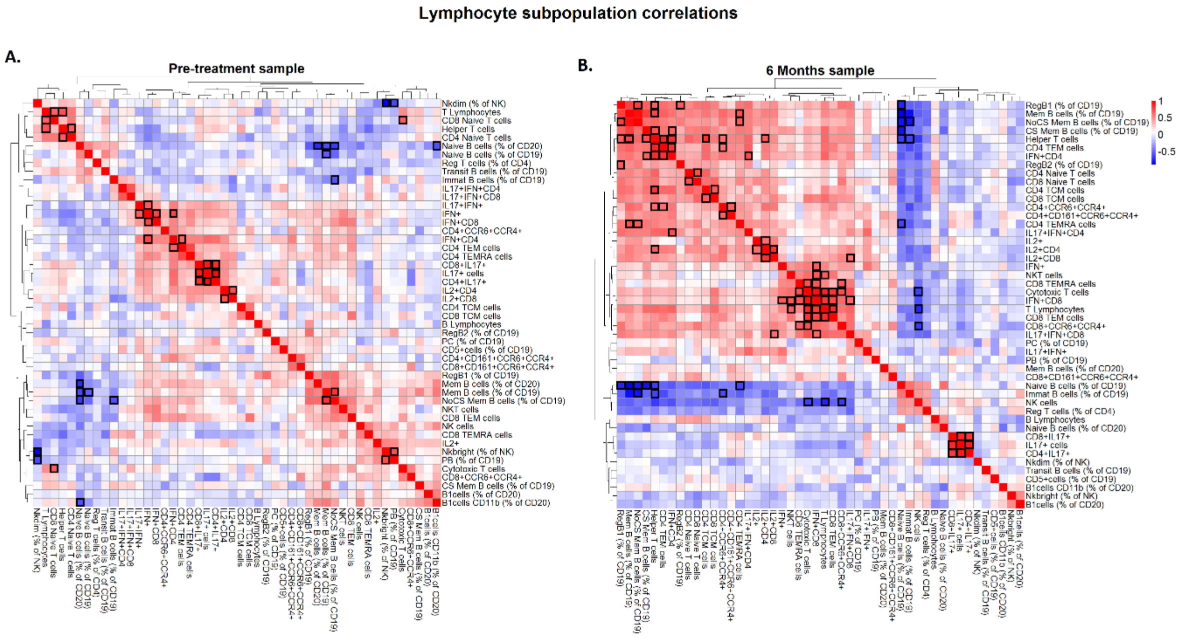

Supplementary Figure S2. Lymphocyte subpopulation correlations.
In A and B, the matrix indicates the coregulation between pairs of cell types in MS patients before (A) and after 6 months of treatment (B). (n=40) (red: positive Rho correlation coefficient, blue: negative Rho correlation coefficient. Remarked points indicate statistically significant correlation p-values <0.05). Unbiased clustering of coefficients was performed to group coregulated cell types. Upon clustering, important differences between the two matrices were observed, indicating the strong effect of fingolimod on the lymphocyte subpopulations. **A**. Four positive clusters were observed with coregulation of helper T cells, CD8 naïve T cells, CD4 naïve T cells and T cells; coregulation of cytokine-producing cells (IFNγ and IL17) and CD4 and CD8 lineages; coregulation of memory B cells and regulatory B cells, and coregulation of IL-2-producing, NK bright, plasmablast and TEMRA CD8 cells. Two negative clusters were observed with coregulation between NK dim cells and plasmablasts and coregulation of naïve B cells and memory and regulatory B cells. **B**. A main positive cluster was observed in which two important regions were emphasized: the coregulation between memory B cells and helper T cells and the coregulation between NKT cells and TEM and TEMRA CD8 T cells. Additionally, another positive cluster was found with the coregulation of IL17-producing cells and CD4 and CD8 lineages. Two negative clusters with coregulation of helper T cells and memory and regulatory B cells and coregulation of NK cells and cytotoxic cells and Cd8+CCR6+CCR4 cells were also found.

Supplementary Figure S3. Heatmap of logistic regression coefficients between lymphocytes subpopulations and several response measures.

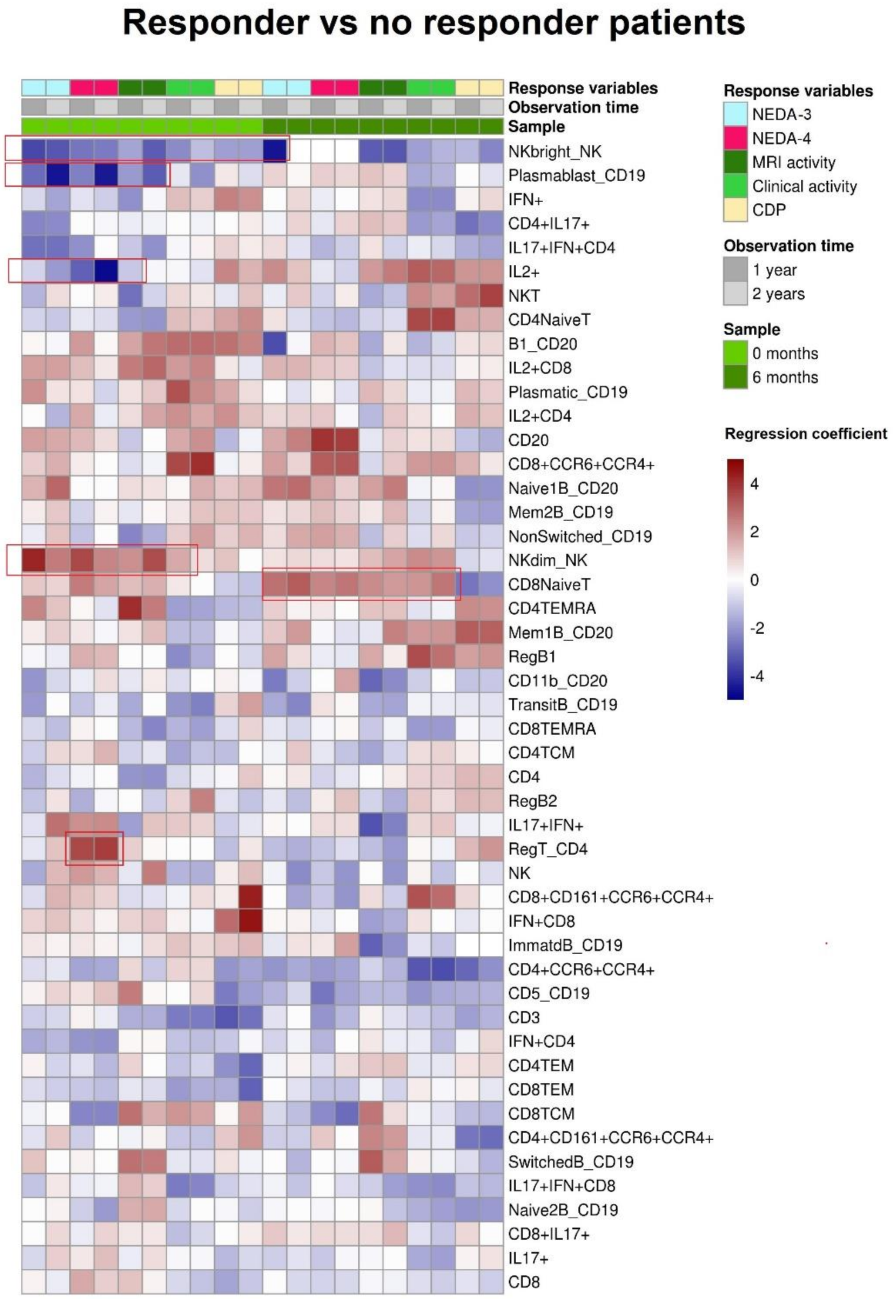

Supplementary Figure S3. Heatmap of logistic regression coefficients between lymphocytes subpopulations and several response measures.

Heatmap of the logistic regression coefficients for each of the lymphocyte subpopulations as predictors for the response variables of interest (NEDA-3, NEDA4, MRI activity, clinical activity and CDP) organized by columns and marked with the color key “Response variables” in first line. These response variables were determined at 1 and 2 years and have been discriminated by columns and marked with the color key “Observation time” in the second line. The lymphocytes subpopulations obtained before and at 6 months of treatment are shown in columns 0-10 (light green) and 11-20 (dark green) respectively as indicated by color key “Sample” in third line. Coefficients are represented using the mean of the posterior distributions, as indicated by the color scale, with red representing a positive correlation with the predictors shown as columns and blue indicating a negative correlation. The subpopulations with the best coefficients for most of the target variables are locked in the red rectangle

Supplementary Figure S4. Differential regulation of S1P, oxidative stress and Th17 pathways by fingolimod.

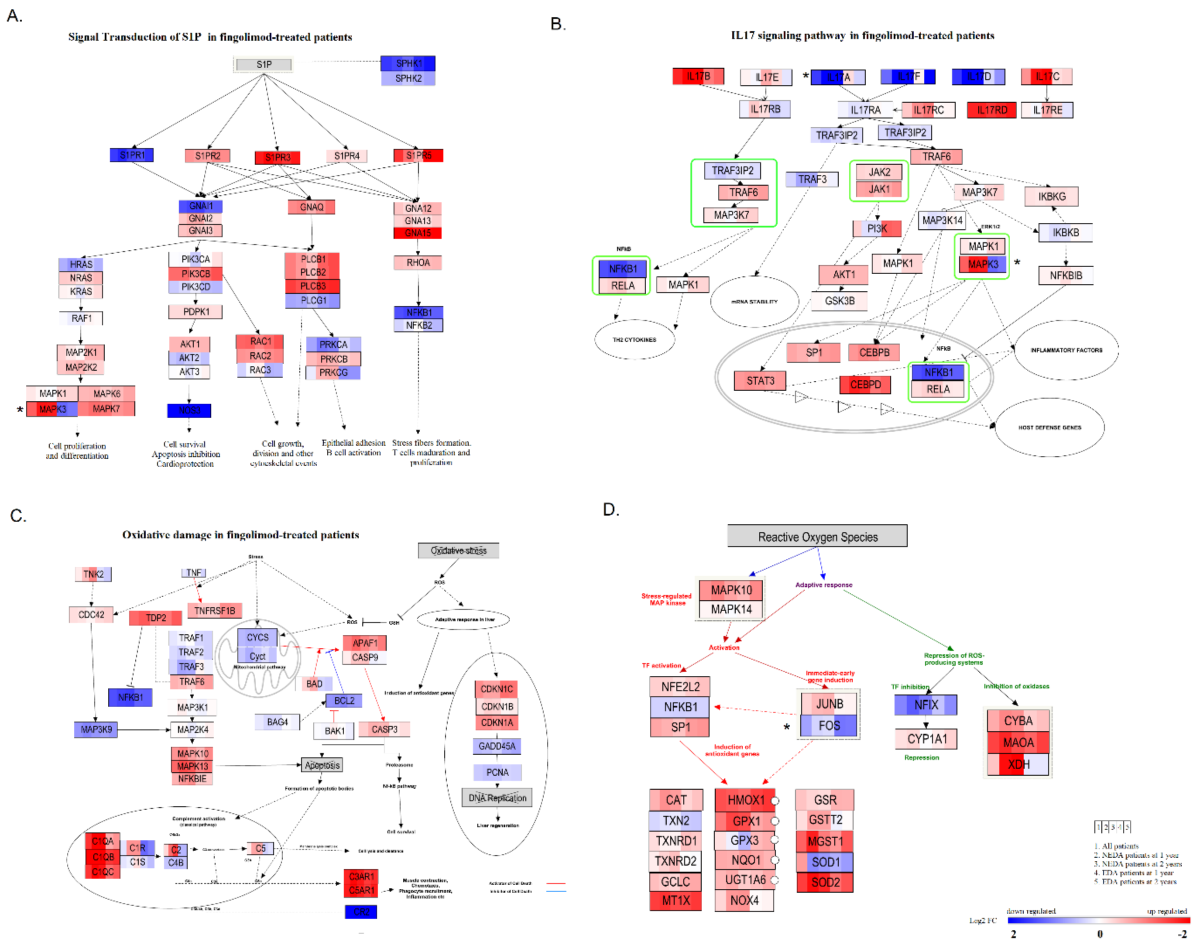

Supplementary Figure S3. Differential regulation of S1P, oxidative stress and Th17 pathways by fingolimod. The gene expression fold-change was mapped in color from red for up-regulated genes to blue for down-regulated genes in response to fingolimod after 6 months of treatment. For each gene, the fold change was calculated for 5 subgroups (all patients, NEDA patients at 1 year, NEDA patients at 2 years, EDA patients at 1 year and EDA patients at 2 years) to assess the differential regulation between R and NR patients. * Genes with significant differences between responder and non-responder patients. Pathways were obtained from wikipaths and drawn using the PathVisio program **A.** S1P pathway (ontology terms: PW0000960). The only gene in this pathway that exhibited differential regulation after 6 months of treatment was MAPK3, which was up-regulated in R patients and down-regulated in NR patients (at 1 and 2 years). **B.** Th17 pathway (ontology terms: PW0000960). IL17-A was down-regulated after 6 months only in R patients. **C- D.** Oxidative stress response pathway (*Homo sapiens*) adapted from wikipaths (ontology terms: PW0000378). The only gene in this pathway that exhibited differential regulation after 6 months of treatment was FOS, which was significantly down-regulated only in NR patients at 2 years.

Supplementary figure S5. Differentially Expressed Genes (DEGs): volcano plots, top 50 genes and PCA.

S4.1. DEGs of samples obtained from naive patients vs MS patients (previously treated with natalizumab or interferon beta) before fingolimod treatment.

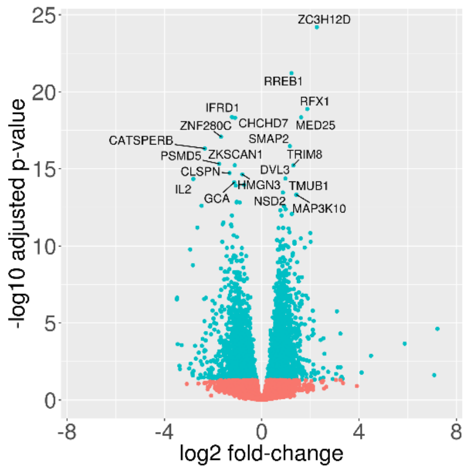

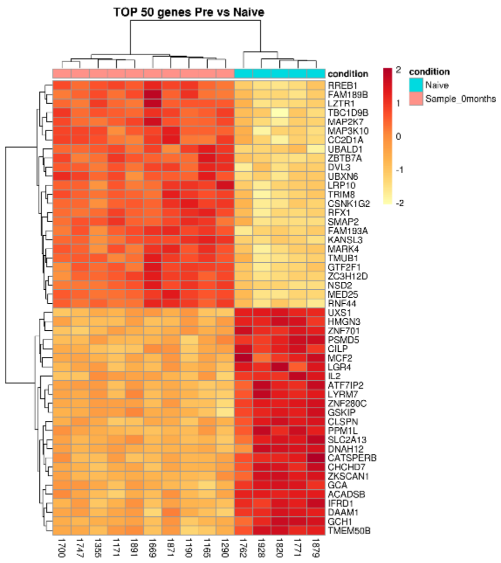

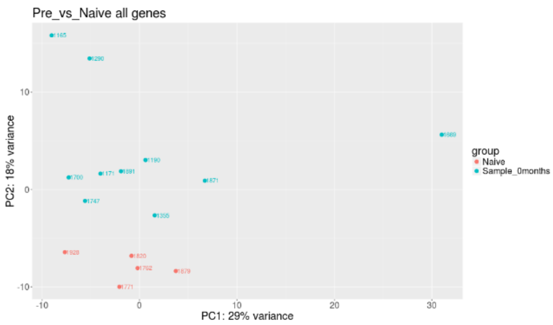

S4.2. DEGs of samples obtained from healthy controls vs MS patients (previously treated with natalizumab or interferon beta) before fingolimod treatment.

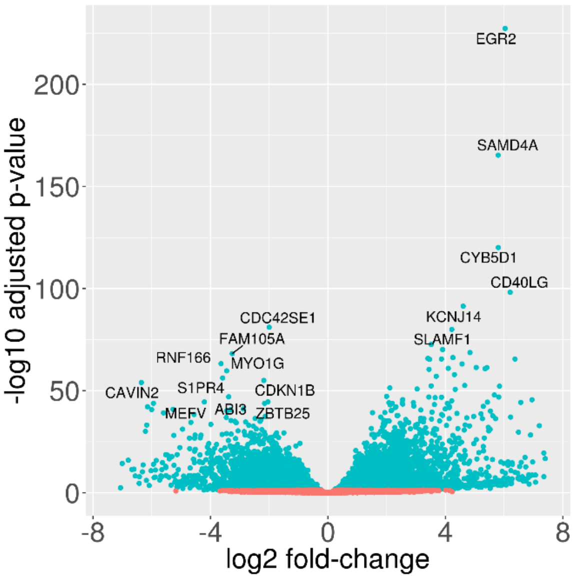

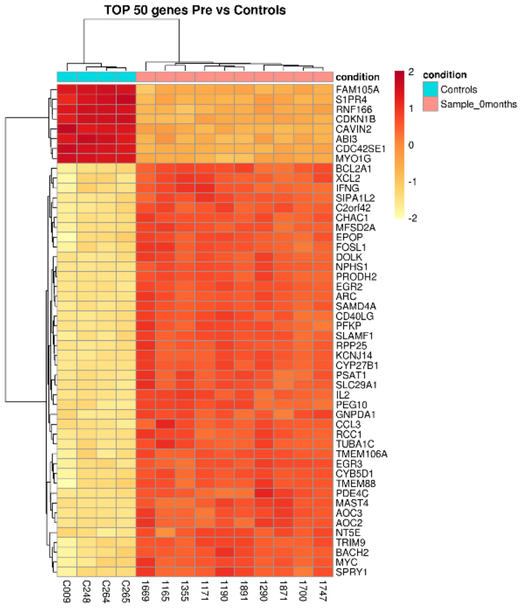

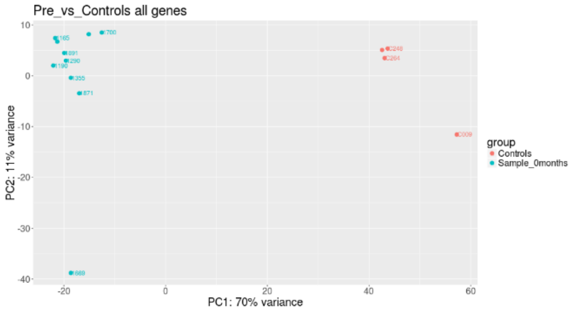

S4.3. DEGs of samples obtained from NTZ-group vs No-NTZ group before fingolimod treatment.

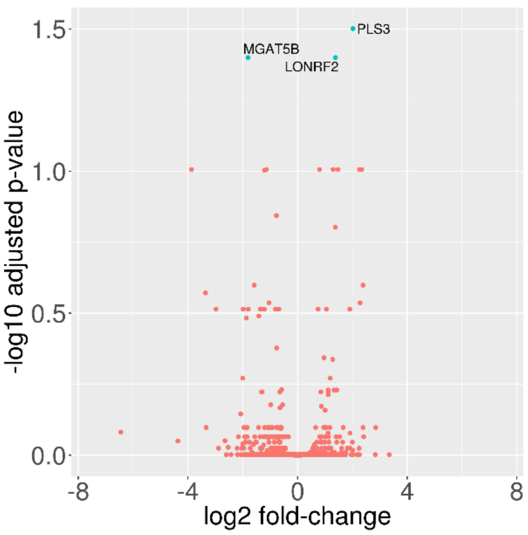

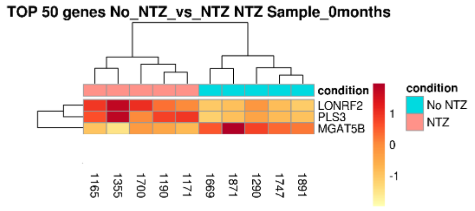

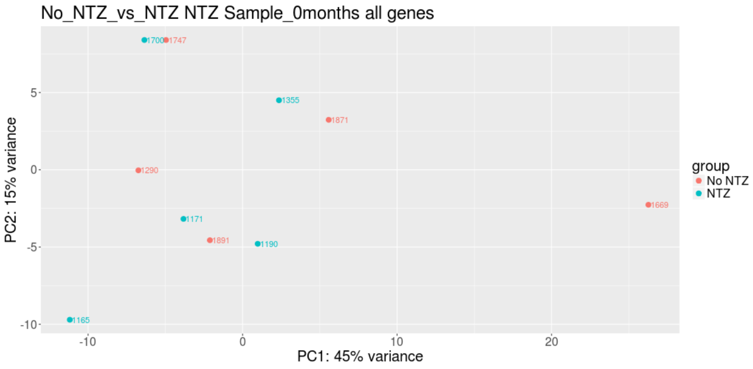

S4.4. DEGs of samples obtained before and after (6 months) fingolimod treatment in all MS patients.

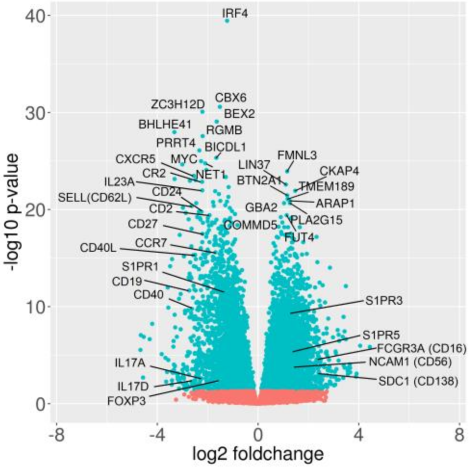

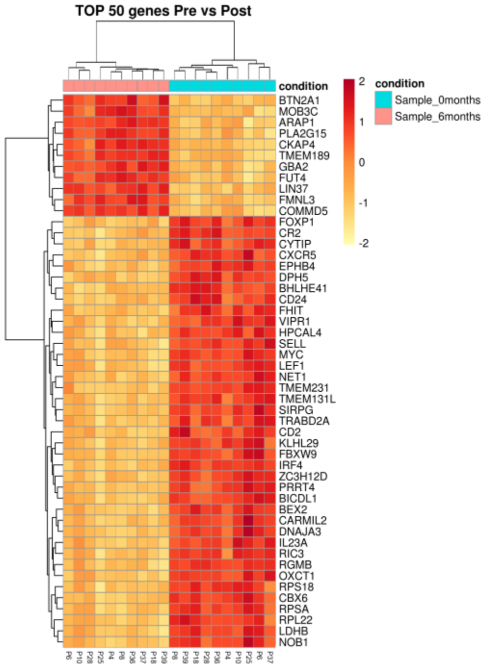

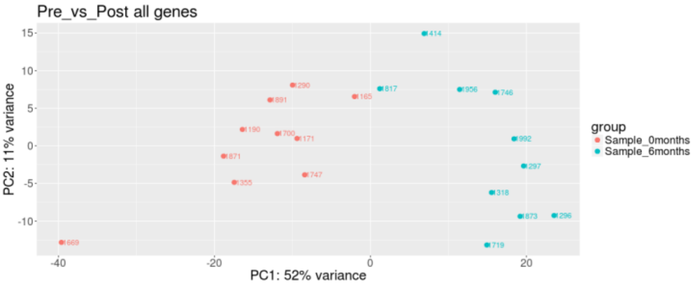

S4.5. DEGs of samples obtained before and after (6 months) fingolimod treatment exclusively in MS patients who achieved NEDA-4 status at 1 year.

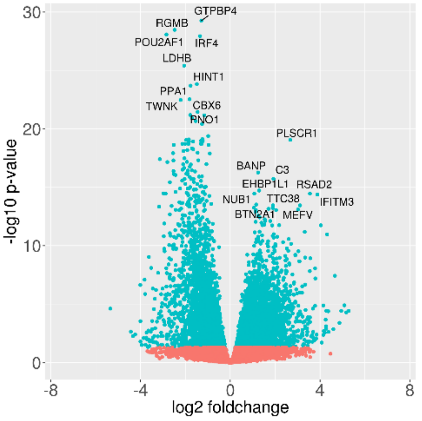

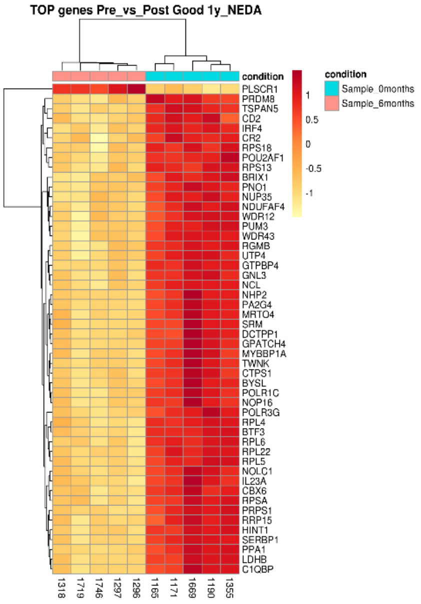

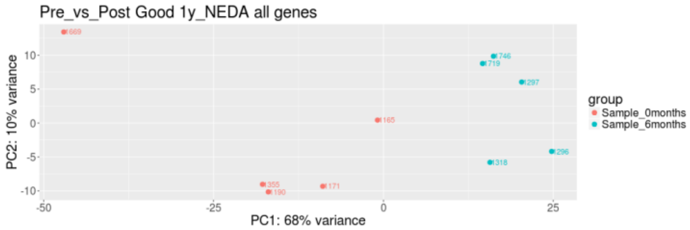

S4.6. DEGs of samples obtained before and after (6 months) fingolimod treatment exclusively in MS patients who did not achieve NEDA-4 status at 1 year.

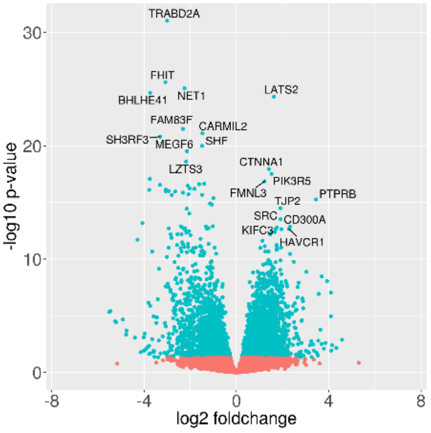

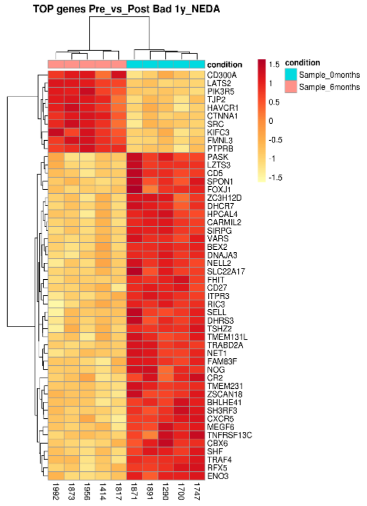

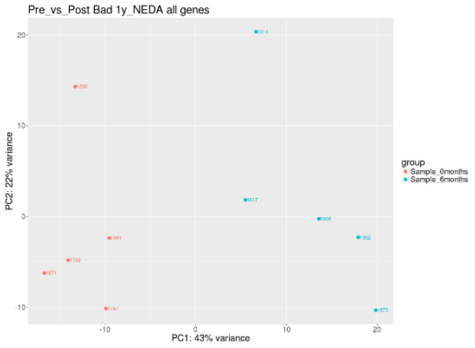

S4.7. DEGs of samples obtained before vs after (6 months) fingolimod treatment in MS patients who achieved NEDA-4 status at 2 years.

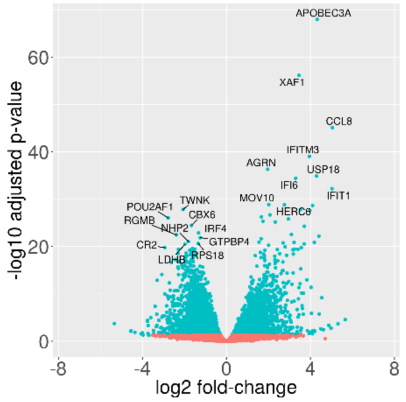

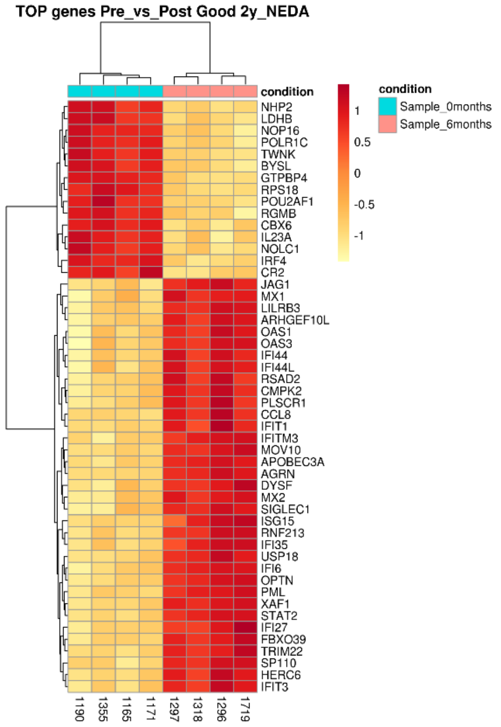

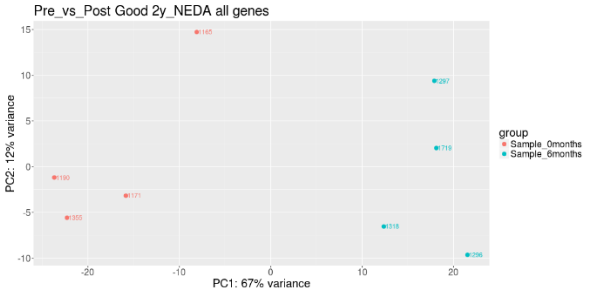

S4.8. DEGs of samples obtained before and after (6 months) fingolimod treatment in MS patients who did not achieve NEDA-4 status at 2 years.

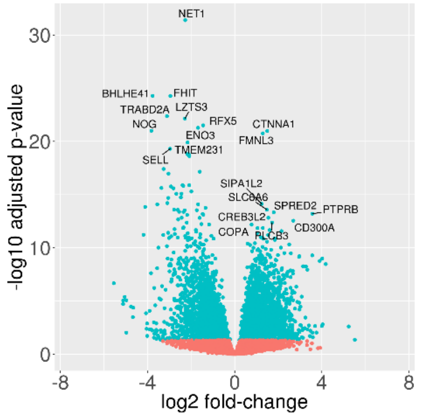

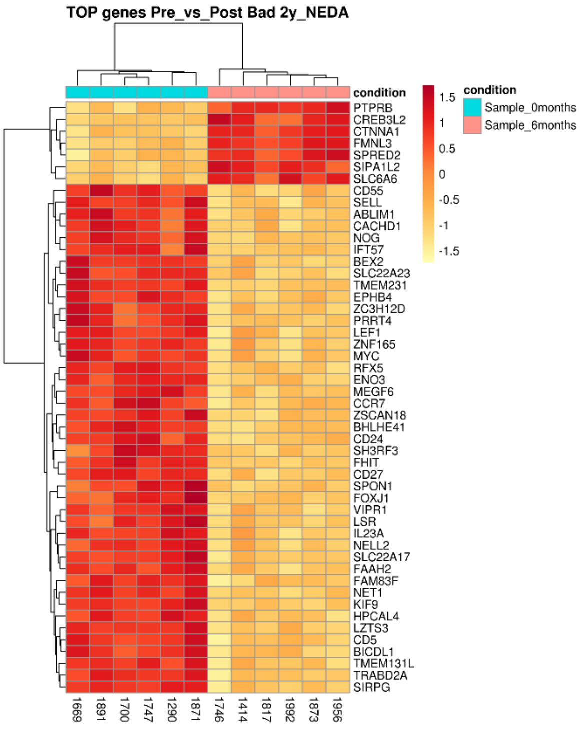

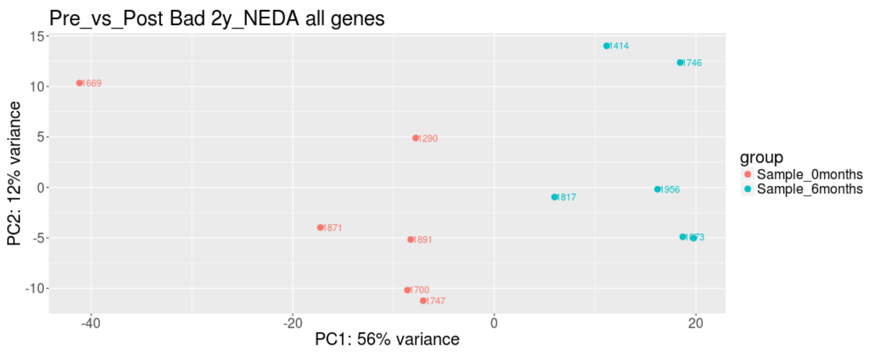

S4.9. DEGs after 6 months of fingolimod in samples obtained from NEDA-4 vs EDA-4 patients when it was calculated at 1 year.

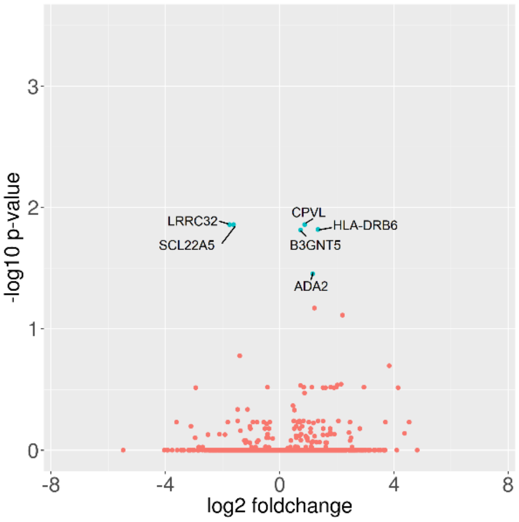

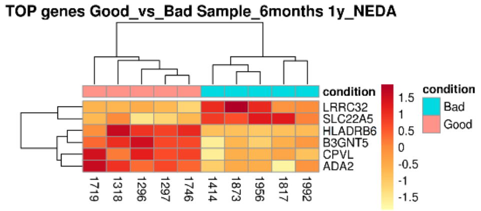

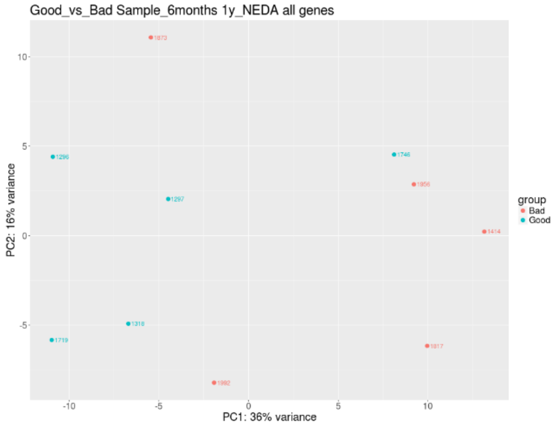

S4.10. DEGs after 6 months of fingolimod treatment in samples obtained from NEDA-4 vs EDA-4 patients when it was calculated at 2 years.

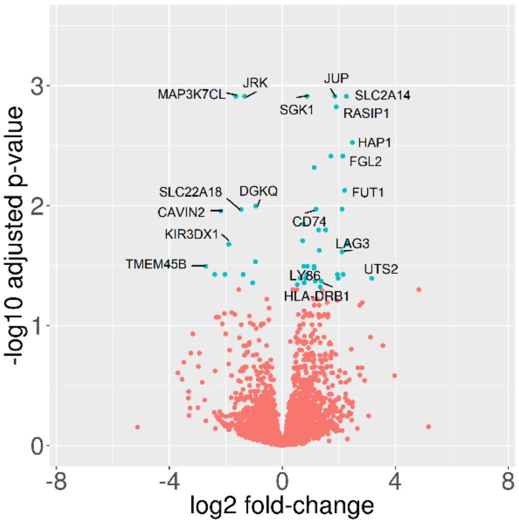

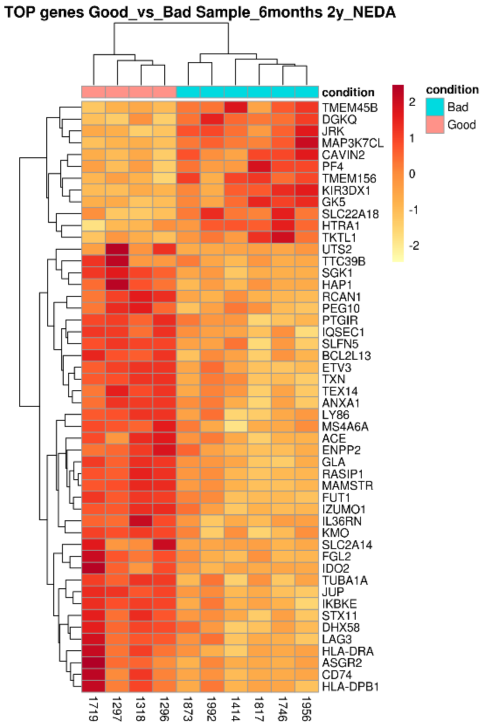

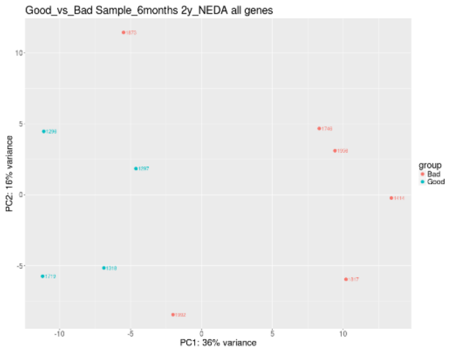

S4.11. DEGs before fingolimod treatment in samples obtained from NEDA-4 vs EDA-4 patients when it was calculated at 1 year.

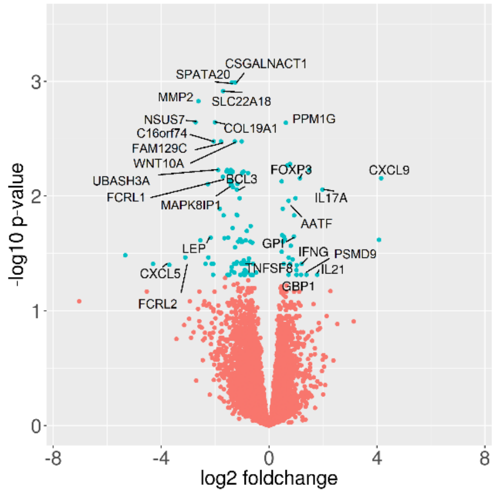

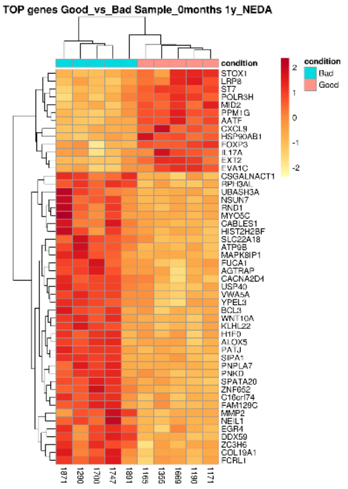

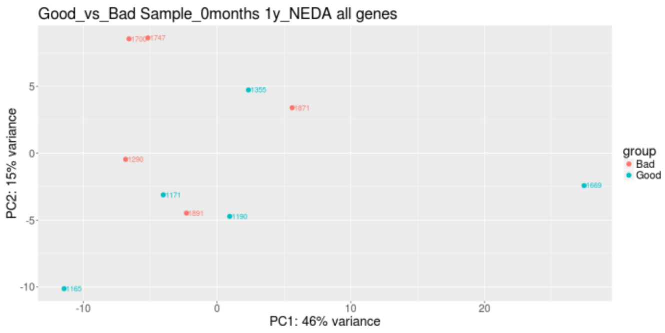

S4.12. DEGs before fingolimod treatment in samples obtained from NEDA-4 vs EDA-4 patients when it was calculated at 2 years.

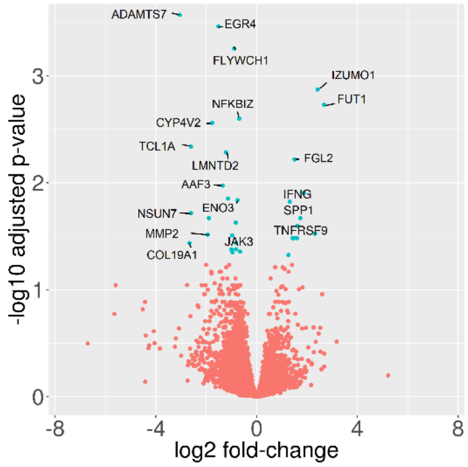

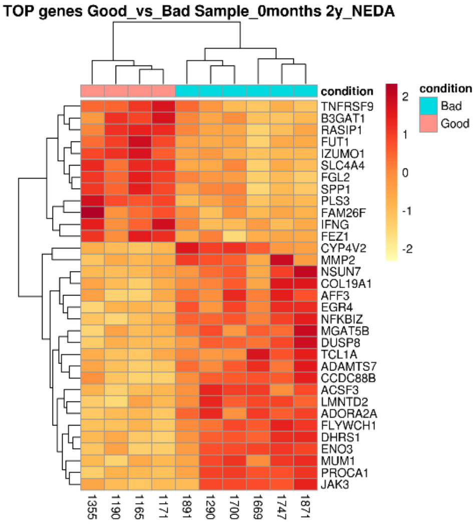

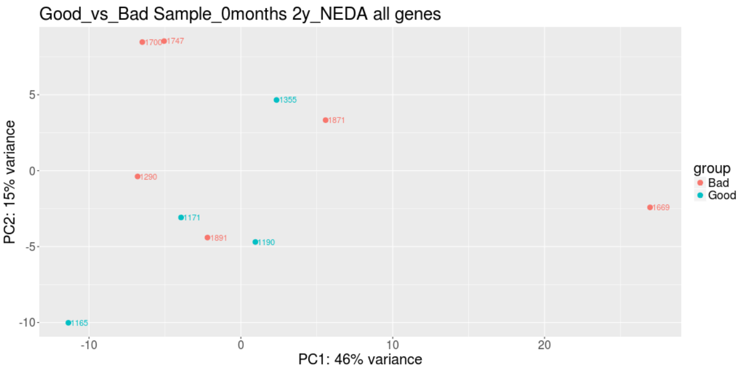

Supplement: Supplementary file 1 [file Data_Sheet_1.PDF]
